# Impact of the fungal pathogen *Fusarium oxysporum* on the taxonomic and functional diversity of the common bean root microbiome

**DOI:** 10.1186/s40793-023-00524-7

**Published:** 2023-08-03

**Authors:** Lucas William Mendes, Jos M Raaijmakers, Mattias de Hollander, Edis Sepo, Ruth Gómez Expósito, Alisson Fernando Chiorato, Rodrigo Mendes, Siu Mui Tsai, Victor J Carrión

**Affiliations:** 1 Cell and Molecular Biology Laboratory, Center for Nuclear Energy in Agriculture CENA, University of Sao Paulo USP, Piracicaba, SP 13416-000 Brazil; 2 Departament of Microbial Ecology, Netherlands Institute of Ecology NIOO-KNAW, Wageningen, 6708 PB The Netherlands; 3 Institute of Biology, Leiden University, Leiden, the Netherlands; 4 Centro de Análises e Pesquisa Tecnológica do Agronegócio dos Grãos e Fibras, Instituto Agronômico IAC, Campinas, 130001-970 Brazil; 5 Laboratory of Environmental Microbiology, Embrapa Environment, Jaguariuna, 18020-000 Brazil; 6 Departamento de Microbiología, Instituto de Hortofruticultura Subtropical y Mediterránea ‘La Mayora’, Universidad de Málaga-Consejo Superior de Investigaciones Científicas (IHSM-UMA-CSIC), Universidad de Málaga, Málaga, Spain

**Keywords:** rhizosphere, endosphere, Metagenome, Metatranscriptome, Plant-microbe interaction

## Abstract

**Background:**

Plants rely on their root microbiome as the first line of defense against soil-borne fungal pathogens. The abundance and activities of beneficial root microbial taxa at the time prior to and during fungal infection are key to their protective success. If and how invading fungal root pathogens can disrupt microbiome assembly and gene expression is still largely unknown. Here, we investigated the impact of the fungal pathogen *Fusarium oxysporum* (*fox*) on the assembly of rhizosphere and endosphere microbiomes of a *fox-*susceptible and *fox-*resistant common bean cultivar.

**Results:**

Integration of 16S-amplicon, shotgun metagenome as well as metatranscriptome sequencing with community ecology analysis showed that *fox* infections significantly changed the composition and gene expression of the root microbiome in a cultivar-dependent manner. More specifically, *fox* infection led to increased microbial diversity, network complexity, and a higher proportion of the genera *Flavobacterium*, *Bacillus*, and *Dyadobacter* in the rhizosphere of the *fox*-resistant cultivar compared to the *fox*-susceptible cultivar. In the endosphere, root infection also led to changes in community assembly, with a higher abundance of the genera *Sinorhizobium* and *Ensifer* in the *fox*-resistant cultivar. Metagenome and metatranscriptome analyses further revealed the enrichment of terpene biosynthesis genes with a potential role in pathogen suppression in the *fox*-resistant cultivar upon fungal pathogen invasion.

**Conclusion:**

Collectively, these results revealed a cultivar-dependent enrichment of specific bacterial genera and the activation of putative disease-suppressive functions in the rhizosphere and endosphere microbiome of common bean under siege.

**Supplementary Information:**

The online version contains supplementary material available at 10.1186/s40793-023-00524-7.

## Introduction

The rhizosphere and root endosphere are hotspots for a myriad of microorganisms that, upon expression of specific functional traits, can provide a range of benefits for the plant, including nutrient acquisition [1, 2], abiotic stress tolerance [3, 4], and protection against pathogens [5–7]. Plants and microbes have co-evolved beneficial relationships and a tightly regulated defense system for protection against diseases [8, 9]. Several rhizospheric and endophytic bacteria are able to prevent pathogen infections by producing antimicrobial compounds or inducing systemic resistance in the host plant [7, 10, 11]. Studies on disease-suppressive soils further revealed that plant protection is conferred by a subset of the microbiota selected from the indigenous soil microbiome following a pathogen attack on the root system [7, 12, 13]. Hence, microbiome assembly and activation of specific beneficial traits prior to, during, or after infection is key to the protective success of the microbiome.

Recent studies indicated that plant domestication [14–17] and plant breeding for disease resistance [18, 19] have affected the assembly of rhizosphere and endosphere microbiomes [20]. Moreover, plant defense also impacts the rhizosphere microbiome composition as was exemplified with mutants disrupted in specific defense pathways [21, 22] and studies on microbiome analyses of crop cultivars with different levels of resistance to a specific pathogen [18, 19, 23]. If and how root pathogens affect microbiome assembly has been much less documented. The study by Chapelle et al. [5] showed that an invading root pathogenic fungus induces stress responses in the rhizobacterial community and the host plant with concomitant shifts in the microbiome resulting in plant protection. Recently, Zhou et al. [98] also showed that the infection of plants by *Fusarium* impacts the associated microbiome by changing the microbiome structure, decreasing diversity and network complexity. However, how the interplay between pathogen infection and plant resistance affects the assembly and gene expression of the root microbiome, i.e. rhizosphere and endosphere, is still poorly understood.

In this study, we investigated the impact of the fungal root pathogen *Fusarium oxysporum* (*fox*) on the assembly of rhizosphere and endosphere microbiomes of a *fox-*susceptible and *fox-*resistant common bean cultivar. Common bean (*Phaseolus vulgaris* L.) is the most important legume crop for low-income farmers in Latin America and Africa and the second in the world [24, 25]. *Fusarium oxysporum* (*fox*) is a major disease of common bean worldwide and the most efficient strategy for its control is the use of resistant cultivars [26]. In resistant cultivars, structural and chemical defense mechanisms restrict pathogen invasions, such as vascular occlusion, tyloses, deposition of additional wall layers, and infusion of phenols and other metabolites [27]. Although *fox*-resistance in common bean has a genetic basis, we previously demonstrated that the *fox*-resistant common bean cultivar has a different rhizosphere microbiome composition than its *fox*-susceptible counterpart with a higher frequency of beneficial rhizobacterial genera [6, 28, 29]. More specifically, the results showed that beneficial taxa such as *Pseudomonas*, *Bacillus*, and *Paenibacillus*, and antifungal traits such as protein secretion systems and biosynthesis of phenazines, rhamnolipids, and colicin V were enriched in the rhizosphere of the *fox*-resistant bean accession. However, our previous community-based analyses were limited to the rhizosphere and performed in the absence of the fungal root pathogen. To provide a more comprehensive understanding of the impact of the fungal root pathogen on the assembly and gene expression of the root microbiome, we integrated 16S rRNA amplicon, metagenomic, and metatranscriptome sequencing to assess taxonomic and functional differences between the root microbiomes of these two common bean cultivars with contrasting levels of *fox* resistance. We hypothesized that assembly and gene expression in the rhizospheric and endophytic microbiomes of the *fox*-resistant common bean cultivar is more responsive to pathogen invasion than the root microbiome of the *fox*-susceptible cultivar.

## Materials and methods

### Inoculum preparation

The soil-borne pathogen *Fusarium oxysporum* f.sp. *phaseoli* (*fox*; FOP IAC 14629), the causal agent of fusarium wilt in common beans, was grown in an aerated 2% malt extract medium. After 9 days of growth at room temperature, the cultures were filtered through miracloth (EMD Millipore, Billerica MA, USA) to remove mycelial mats. Microconidia left in the filtrate were pelleted by centrifugation at 5000 x g for 10 min and washed twice with 0.01 M MgSO_4_.7H_2_O (Mgsol). The conidial density of the *fox* was determined by direct observation using a haemocytometer and adjusted to a final concentration of 10^7^ conidia mL^− 1^.

### Bioassay and experimental design

Soil samples were collected in an agricultural field at Vredepeel, The Netherlands (0–30 cm depth, 51º32’25.8” N and 5º51’15.1” E). This soil, classified as Gleyic Podzol soil, is an arable agricultural field since 1955, being in the last years cropped with potato and rye (2010), carrot (2011), and maize and rye (2012–2014) under normal agricultural practices [30]. This soil presents pH 5.4, organic matter (OM) content of 3.7%, total N of 970 mg kg^− 1^, available P of 4.6 mg kg^− 1^, and available K of 209 mg kg^− 1^ (Supplementary Table 1). In a previous experiment [6] we screened four common bean cultivars with different levels of resistance against the pathogen *fox*. For the mesocosm experiments of this present study, we used the two most contrasting common bean (*Phaseolus vulgaris* L.) cultivars with different levels of genetic resistance to the root pathogen *fox*, namely *fox*-resistant IAC Milenio [31] and the susceptible IAC Alvorada [32]. Firstly, the plants were pre-germinated in a mixture of potting soil soil/sand (1/1) at 25ºC for 10 days. After this period the plants were carefully taken from the trays and their roots were washed in tap water. The clean roots were then immersed in a conidial suspension of the *fox* at a density of 10^7^ conidia mL^− 1^ for 1 min, and the plants were transplanted to PVC pots containing approximately 2 kg of soil. Control pots were used without the pathogen inoculation, and pots without plants were considered bulk soil. In each pot, two plants were transplanted and continued to grow at 25ºC (day/night) with 12 h photoperiod. Temperature and moisture were regularly adjusted to create optimal growth conditions for the plants. In total, 30 pots [6 bulk soil + (2 common bean cultivars x 2 treatments x 6 replicates)] were used in the experiments. The plants were grown for approximately 35 days after transplantation.

### Rhizosphere and endosphere sampling

The plants were collected at the R1 stage (early flower) and the roots were shaken to remove the loose soil and the firmly attached soil, considered to be rhizosphere soil, was collected with a sterile spatula. The soil samples collected from the pots without plants were considered bulk soil. The bulk soil and rhizosphere samples were stored at -80ºC until further processing. After rhizosphere sampling, the remaining roots were used for the extraction of the endophytic community. Firstly, the roots were sterilized by washing with Mgsol (MgSO_4_.7H_2_O) containing 0.01% (vol/vol) Tween 20, followed by two rinses with Mgsol. Roots were immersed for 2 min under slow agitation in 1% bleach solution containing 0.01% (vol/vol) Tween 20 and rinsed five times with Mgsol. As a final step, the roots were rolled on Luria-Bertani (rich media) agar plates to verify root surface sterilization. Roots that showed no bacterial growth were used for further analysis. The plant infection was confirmed based on visual symptoms and plating of the fragment roots in the PDA medium (Supplementary Fig. 1). For the plant and microbial cell separation, we follow the protocol described by Chapelle et al. (2016) with some modifications. Briefly, root tissues were disrupted using a blender in a known volume of Mgsol, and the homogenate was filtered through 25-µm miracloth (EMD Millipore). The flow-through was further cleaned by centrifugation at 500 g for 10 min, and the bacterial cells were collected by centrifuging the resulting supernatant at 9500 rpm for 15 min. The pellet, consisting of endophytic microbes contaminated with plant material, was suspended in 3.5 ml Mgsol buffer supplemented with Nycodenz® resin (PROGEN Biotechnik, Germany) to a final concentration of 50% w/v. A Nycodenz density gradient was mounted above the sample by slowly depositing various layers of Nycodenz (3 ml of 35% Nycodenz, 2 ml of 20% Nycodenz, 2 ml of 10% Nycodenz) and the gradient was centrifuged for 45 min at 8500 rpm in swinging bucket rotor Sorvall HB-6 (Thermo Scientific, Waltham, EUA). Endophytic microbial cells, appearing as an opalescent whitish band, were recovered by pipetting. The recovered thin layer was washed five times with Mgsol and centrifuged at 13,000 g for 5 min to remove the Nycodenz resin. Finally, bacterial cells were suspended in 500 µl of Mgsol, recovered by quick centrifugation (16,000 g in a table-top centrifuge), frozen in liquid nitrogen, and stored at -80 °C.

### DNA and RNA extraction and sequencing

DNA and RNA extraction from bulk soil and rhizosphere samples was carried out using the RNA PowerSoil® Total RNA Isolation Kit along with RNA PowerSoil® DNA Elution Accessory Kit (MoBio Laboratories, Carlsbad, CA, USA), according to the manufacturer’s protocol. DNA extraction of endophytic cells was carried out in six replicates using the Meta-G-Nome™ DNA Isolation Kit (Epicentre, Madison, WI, USA) according to the manufacturer’s protocol. Measurements of DNA quality and quantity were performed by 1% sodium boric acid [33] agarose gel electrophoresis, and NanoDrop 1000 spectrophotometry (Thermo Scientific, Waltham, EUA). For taxonomical profiling of the bacterial communities, a total of 54 DNA samples (6 bulk soil, 24 rhizosphere, and 24 endosphere) targeting the V3-V4 region of the 16 S rRNA gene were sequenced (Baseclear, Leiden, The Netherlands) on an Illumina Miseq Sequencing System (Illumina, San Diego CA, USA) according to the company’s protocol. For shotgun metagenome, a total of 54 DNA samples (6 bulk soil, 24 rhizosphere, and 24 endosphere) were sequenced (Erasmus MC Center for Biomics, Rotterdam, The Netherlands) on an Illumina Hiseq PE 2 × 300 (Illumina) according to the company’s protocol. For metatranscriptomics, 30 RNA samples (6 bulk soil and 24 rhizosphere) were sequenced (Erasmus MC) on an Illumina Hiseq PE 2 × 300 (Illumina) according to the company’s protocol.

### 16S rRNA processing and annotation

The data obtained by the 16S rRNA sequencing was analyzed with bioinformatics tools as follows. Initially, primer sequences were removed from the per sample FASTQ files using Flexbar version 2.5 [34]. All reads were trimmed to a minimum length of 150 bp and at least a Phred score of 25 by using fastq-mcf (http://code.google.com/p/ea-utils). The remaining sequences were converted to FASTA format and concatenated into a single file. All reads were clustered into OTUs using the UPARSE strategy by dereplication, sorting by the abundance with at least two sequences, and clustering using the UCLUST smallmem algorithm [35]. These steps were performed with VSEARCH version 1.0.10 [100], which is an open-source and 64-bit multithreaded compatible alternative to USEARCH. Next, chimeric sequences were detected using the UCHIME algorithm [36] implemented in VSEARCH. All reads were mapped before the dereplication to OTUs using the usearch_global method implemented in VSEARCH to create an OTU table and converted to BIOM-Format 1.3.1 [37]. Finally, taxonomic information for each OTU was added to the BIOM file by using the RDP Classifier version 2.10 [38]. Singletons and doubletons, mitochondrion, chloroplast, and eukaryotic sequences were removed and the BIOM file generated was used for statistical analyses. The 16S rRNA data are available at NCBI SRA under the identification PRJNA904225.

### Metagenome and metatranscriptome data processing

For the metagenome and metatranscriptome data, the paired-end reads were trimmed with the sliding window approach used by Sickle [39] to keep reads with at least a Phred score of 30 and 150 base pairs in length. Contamination of reads originating from the host plant was removed by mapping with Bowtie 2.2.5 [40] in very sensitive mode against the draft genome of *Phaseolus vulgaris*, and paired and unpaired data were stored separately. Reads of each treatment were pooled together for an assembly with Megahit [41] using kmers with lengths 33, 55, 77, 99, and 127 and the careful flag enabled. On the resulting contigs, genes were predicted with Prodigal 2.61 [42] in metagenomics mode and stored in General Transfer Format using Cufflinks 2.1.1 [43]. Genes were assigned to taxonomy by running Diamond 0.7.9 [44] against the non-redundant blast database of the NCBI from 20150311. The lowest common ancestor classification was determined using MEGAN 5.10 [45] by taking the top 50% hits and filtering them for a minimum score of 50 and maximum expected value of 0.01 and converting the gene identifiers to taxonomy ids using the mapping provided by MEGAN. For functional annotation, UProc [46] was used to annotate genes with KEGG release 20140317 [47], COG release 2014 [48], and Pfam 28 [49]. Also, Biosynthesis Gene Clusters (BGCs) were annotated using antiSMASH 3.0 [50]. An abundance table was created by first mapping all reads to the contigs with BamM [99], which uses samtools 1.2 [51] and bwa-mem 0.7.12 [52] followed by counting all number of reads mapping to a contig with featureCounts [53]. The metagenome and metatranscriptome data are publicly available at NCBI SRA under the identifications PRJNA904562 and PRJNA904281, respectively.

### Data analysis and statistics

To compare the microbial community structure between the treatments, we used a cumulative-sum scaling (CSS) method to avoid the biases generated by current sequencing technologies due to uneven sequencing depth [54]. With the normalized OTU table, we calculated the Bray-Curtis dissimilarity matrix and used it to build Constrained Principal Coordinate Analysis (CAP) constrained by Phylogenetic Group using the function *capscale* retrieved from Vegan v.2.3-2 package [55] and implemented in the Phyloseq package v.1.10 [56], both in R. We used permutational multivariate analysis of variance PERMANOVA [57] to test whether sample categories harbored significantly different microbial community structures using Past 3 software [58]. For alpha diversity, the OTU table based on 16S rRNA sequencing was rarefied to counts up to 48,000 reads using the command *alpha_rarefaction.py* available in Qiime [59]. This was the lowest sequencing depth obtained from a sample. To calculate the diversity indexes, we used the *alpha_diversity.py* command, obtaining Observed OTUs, Shannon, and Chao1 metrics. One-way ANOVA and Tukey HSD were performed in R. Also, to have a better understanding of the microbial community assembly in each niche, we calculated several species abundance distribution models and determined whether neutral or niche-based mechanisms drove the communities. We hypothesized that bulk soil would be driven by neutral-based processes, while the rhizosphere and endosphere would respond to niche-based processes. For this, we used the command Radfit from the R package vegan to evaluate several abundance models and a zero-sum multinomial (ZSM) model using TeTame [60]. Species abundance distribution models were compared based on the Akaike Information Criterion weight calculated as previously reported [1, 61]. In addition, to better understand the effect of the pathogen infection on the community we tested the niche occupancy by classifying the OTUs into specialist and generalist. The niche occupancy was verified by the multinomial species classification method ‘clamtest’ available in the ‘vegan’ package. This method compares the microbial abundance between two habitats and classifies the group of species that are similarly distributed across both habitats as generalist, and classifies as specialist the species more abundant in one habitat compared to the other [62]. For CLAM analysis, it was considered a significant level for individual test of alpha 0.005 and a specialization threshold of 0.66.

To compare the differential abundance of groups between the treatments we conducted an LDA Effect Size (LEfSe) analysis, according to Segata et al. [63]. For this, the analysis first uses the non-parametrical factorial Kruskal-Wallis sum-rank test [64] to detect features with significant differential abundance; then, the biological significance is subsequently investigated using a set of pairwise tests using unpaired Wilcoxon rank-sum test [65]; finally, LEfSe uses LDA [66] to estimate the effect size of each differentially abundant feature. We applied the LEfSe analysis for the three datasets, i.e. 16S rRNA, metagenome, and metatranscriptome to detect features commonly different among them.

In addition, network analyses were performed to assess the complexity of the interactions among microbial taxa. Non-random co-occurrence analyses were performed using SparCC [67]. For this, the 500 most abundant OTUs per treatment were retained for analysis, representing > 98% of the sequences. For each network analysis, *P*-values were obtained by 99 permutations of random selections of the data table, subjected to the same analytical pipeline. SparCC correlations with a magnitude > 0.7 or < -0.7 and statistically significant (*P* < 0.01) were included into network analyses. The nodes in the reconstructed networks represent the OTUs at 97% identity, whereas the edges (that is, connections) correspond to a strong and significant correlation between nodes. The topology of the network was calculated based on a set of measures, including the number of nodes and edges, modularity, the number of communities, average path length, network diameter, averaged degree, and clustering coefficient [68, 69]. Co-occurrence analyses were carried out using the Python module ‘SparCC’ and network visualization and properties were constructed using the interactive platform Gephi [70]. The code used in the analysis can be found at Zenodo (10.5281/zenodo.7447085).

## Results

### Progressive change in microbiome composition moving from soil to rhizosphere and endosphere

After quality trimming, approximately 3.5 million 16S rRNA sequences were obtained and we identified 5,298 prokaryotic operational taxonomic units (OTUs) at 97% sequence similarity. For metagenome and metatranscriptome, we obtained approximately 388 and 111 million quality sequences, respectively. Taxonomic classification of the 16S OTUs at the phylum level highlighted that bulk soil samples were dominated by Proteobacteria (36.2% of sequences), followed by Actinobacteria (19.2%), Acidobacteria (15.9%), Firmicutes (7.6%), Bacteroidetes (5.4%), and Gemmatimonadetes (4.2%). The rhizosphere samples were dominated by Proteobacteria (39.5%), followed by Actinobacteria (19.4%), Acidobacteria (12.2%), Bacteroidetes (9.6%), Gemmatimonadetes (4.2%), and Candidatus Saccharibacteria (3.5%). However, the endophytic community was almost totally represented by Proteobacteria (97.9%), where 98.5% of these OTUs belong to the Alphaproteobacteria class. A small proportion of sequences were classified into archaeal phyla (< 0.1%). The taxonomic composition of the bacterial microbiome was similar among the three datasets obtained, i.e. 16S rRNA, metagenome, and metatranscriptome (Supplementary Fig. 2).

**Fig. 1:**
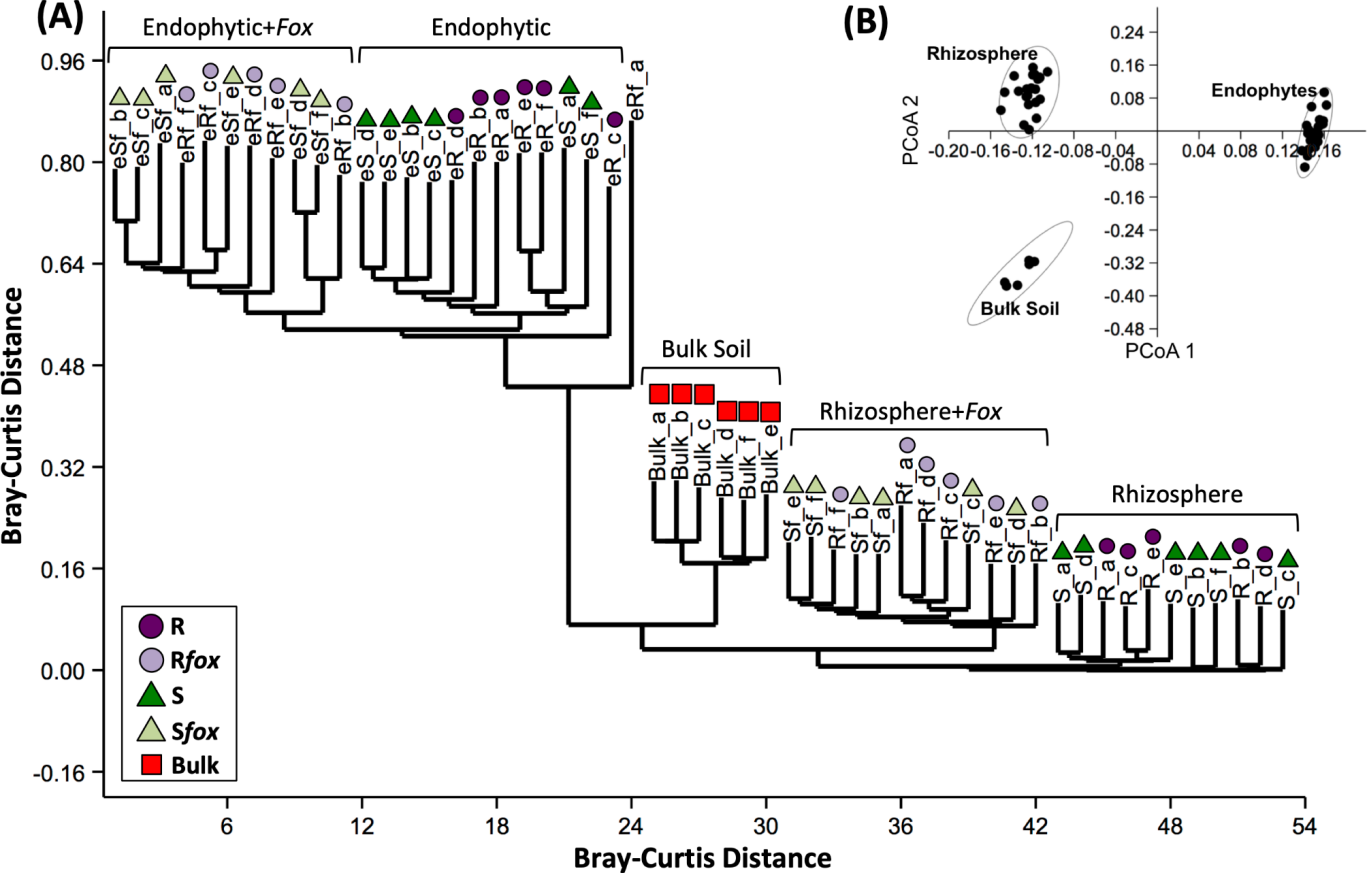
Microbiome composition comparative analysis, based on 16S rRNA profiling, for bulk soil, rhizospheric, and endosphytic communities non-inoculated or inoculated with *Fusarium oxysporum* (*fox*). **(A)** Dendogram analysis based on Bray-Curtis similarity distance. **(B)** Principal Coordinate Analysis (PCoA). R = *fox*-resistant cultivar; R*fox* = *fox*-resistant cultivar inoculated with *fox*; S = susceptible cultivar; S*fox* – susceptible cultivar inoculated with *fox*; Bulk = bulk soil

To compare the bacterial community structure between the three niches (i.e. bulk soil, rhizosphere, and endosphere), the abundance matrix of taxonomy was converted to the Bray-Curtis distance matrix and used in the analysis. The PCoA analysis showed that the samples were primarily clustered according to the niche (PERMANOVA, *P* = 0.0001); the cluster analysis showed a clear separation between niches and treatments (*P* = 0.0001) (Fig. 1 and Supplementary Table 2). The LDA effect size analysis comparing the microbiome composition based on the three datasets (16S, metagenome, and metatranscriptome) highlighted an over-abundance of Bacteroidetes, Verrucomicrobia, Candidatus Saccharibacteria, Proteobacteria, Chlorobi, and Armatimonadetes in the rhizosphere compared to bulk soil (*P* < 0.05). In the endosphere niche, only Proteobacteria was significantly more abundant as compared to bulk soil (*P* < 0.05) (Supplementary Fig. 3). Collectively these results revealed a shift in community structure and composition at the common bean root-soil interface, which progressively differentiated from the bulk soil to the rhizosphere and endosphere.

### Neutral and niche processes are governing the common bean microbiome assembly

**Fig. 2:**
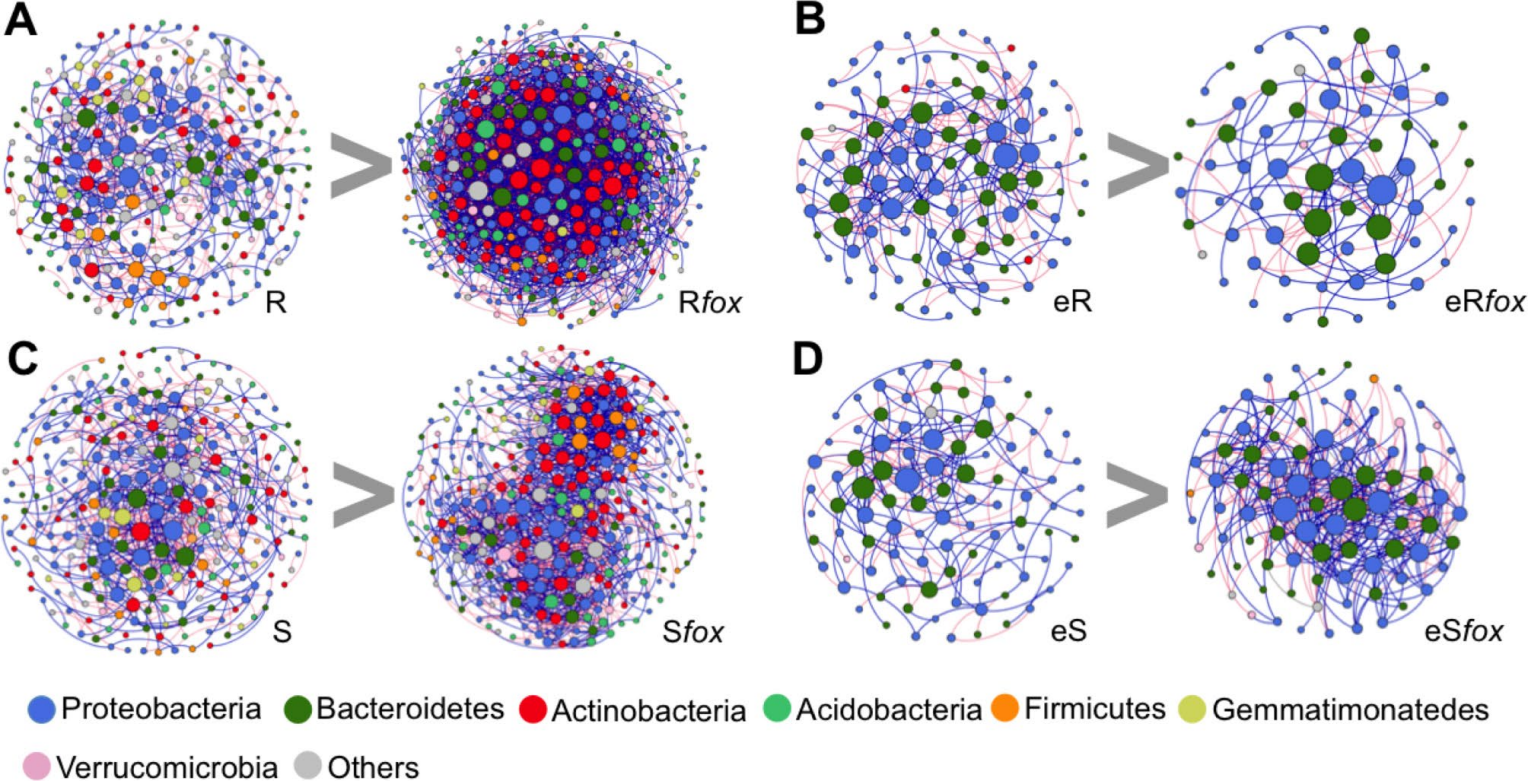
Co-occurrence network analysis of bacterial communities in bulk soil, rhizosphere, and endosphere of common beans non-inoculated or inoculated with *Fusarium oxysporum* (*Fox*). A connection stands for SparCC correlation with magnitude > 0.6 (positive correlation – blue edges) or < -0.6 (negative correlation – red edges) and statistically significant (*P* < 0.01). The size of each node is proportional to the number of connections (that is, degree). Each node represents taxa at OTU label, but labelled at the phylum level. R = Rhizosphere Resistant; R*fox* = Rhizosphere resistant inoculated with *fox*; eR = Endophytic resistant; eR*fox* = Endophytic resistan inoculated with *fox*; S = Rhizosphere susceptible; S*fox* = Rhizosphere susceptible inoculated with *fox*; eS = Endophytic susceptible; eS*fox* = Endophytic susceptible with *fox*

To further dissect these differences in microbiome composition and assembly between the three niches, the abundance of the read counts was fitted to several species abundance distribution (SAD) models. Comparisons based on Akaike’s Information Criterion (AIC) weight allowed us to find the best-fit value from six models. The results showed that the microbial assembly in the bulk soil and endosphere is explained by neutral process (lognormal and ZSM, respectively). On the other hand, the community assembly in the rhizosphere was explained by niche-based process (Mandelbrot) (Supplementary Fig. 4). We then used SparCC correlations to construct co-occurrence networks to verify the response of the microbiome to pathogen infection in the rhizosphere and endosphere of both common bean cultivars. The network reconstruction was markedly different between the treatments, showing an increased complexity in the rhizosphere microbiome after pathogen infection (Fig. 2A and C; Table 1). Interestingly, the rhizosphere network of the resistant cultivar infected with *fox* presented more complexity (nodes = 363, edges = 1781, average degree = 9.813) compared to the susceptible (nodes = 334, edges = 1513, av. degree = 9.060). In the endosphere, the network dynamics was less complex compared to rhizosphere, with a distinct response between the cultivars: the *fox*-resistant decreased the network complexity after infection (nodes = 67, edges = 103, av. degree = 3.07) while the susceptible cultivar exhibited an increased network complexity (nodes = 90, edges = 391, av. degree = 8.68) (Fig. 2B and D; Table 1). The phyla Proteobacteria, Bacteroidetes, and Actinobacteria presented the highest number of correlations in all networks (Supplementary Table 3). The key groups that responded to the pathogen infection in both rhizosphere and endosphere, depicted here as the nodes with a higher number of correlations and higher betweenness centrality [71], were affiliated to the bacterial families Flavobacteriaceae (*Flavobacterium* and *Chryseobacterium*), Cytophagaceae (*Dyadobacter*), Comamonadaceae, Pseudomonadaceae (*Pseudomonas*), and Oxalobacteriaceae (Supplementary Table 4).

**Table 1:** Correlations and topological properties of common bean rhizosphere, endosphere and soil microbiome networks

	Network properties	Resistant	Resistant *Fox*	Susceptible	Susceptible *Fox*	Bulk Soil
**Rhizosphere**	Number of nodes^a^	270	363	288	334	241
	Number of edges^b^	635	1781	646	1513	1527
	Positive edges^c^	320	957	317	899	871
	Negative edges^c^	315	824	329	614	656
	Modularity^e^	37.78	6.133	-140.83	2.33	2.597
	Number of communities^f^	70	44	58	56	37
	Network diameter^g^	7	9	8	11	7
	Average path length^h^	2.37	2.84	2.62	2.98	2.42
	Average degree^i^	4.704	9.813	4.486	9.060	12.672
	Average clustering coefficient^j^	0.114	0.139	0.090	0.148	0.174
**Endosphere**	Number of nodes	109	67	98	90	
	Number of edges	237	103	174	391	
	Positive edges	131	68	107	226	
	Negative edges	106	35	67	165	
	Modularity	3.70	1.38	2.07	2.41	
	Number of communities	29	22	31	12	
	Network diameter	6	4	6	5	
	Average path length	2.22	1.58	2.37	2.18	
	Average degree	4.34	3.07	3.55	8.68	
	Average clustering coefficient	0.158	0.123	0.082	0.222	

aMicrobial taxon (at genus level) with at least one significant (P < 0.01) and strong (SparCC > 0.9 or < − 0.9) correlation. bNumber of connections/correlations obtained by SparCC analysis. cSparCC-positive correlation (> 0.9 with P < 0.01). dSparCC-negative correlation ( < − 0.9 with P < 0.01). eThe capability of the nodes to form highly connected communities, that is, a structure with high density of between nodes connections (inferred by Gephi). fA community is defined as a group of nodes densely connected internally (Gephi). gThe longest distance between nodes in the network, measured in number of edges (Gephi). hAverage network distance between all pair of nodes or the average length off all edges in the network (Gephi). iThe average number of connections per node in the network, that is, the node connectivity (Gephi). jHow nodes are embedded in their neighborhood and the degree to which they tend to cluster together (Gephi)

### Rhizosphere microbiome respond to the pathogen invasion

#### Changes in rhizosphere bacterial community composition

**Fig. 3:**
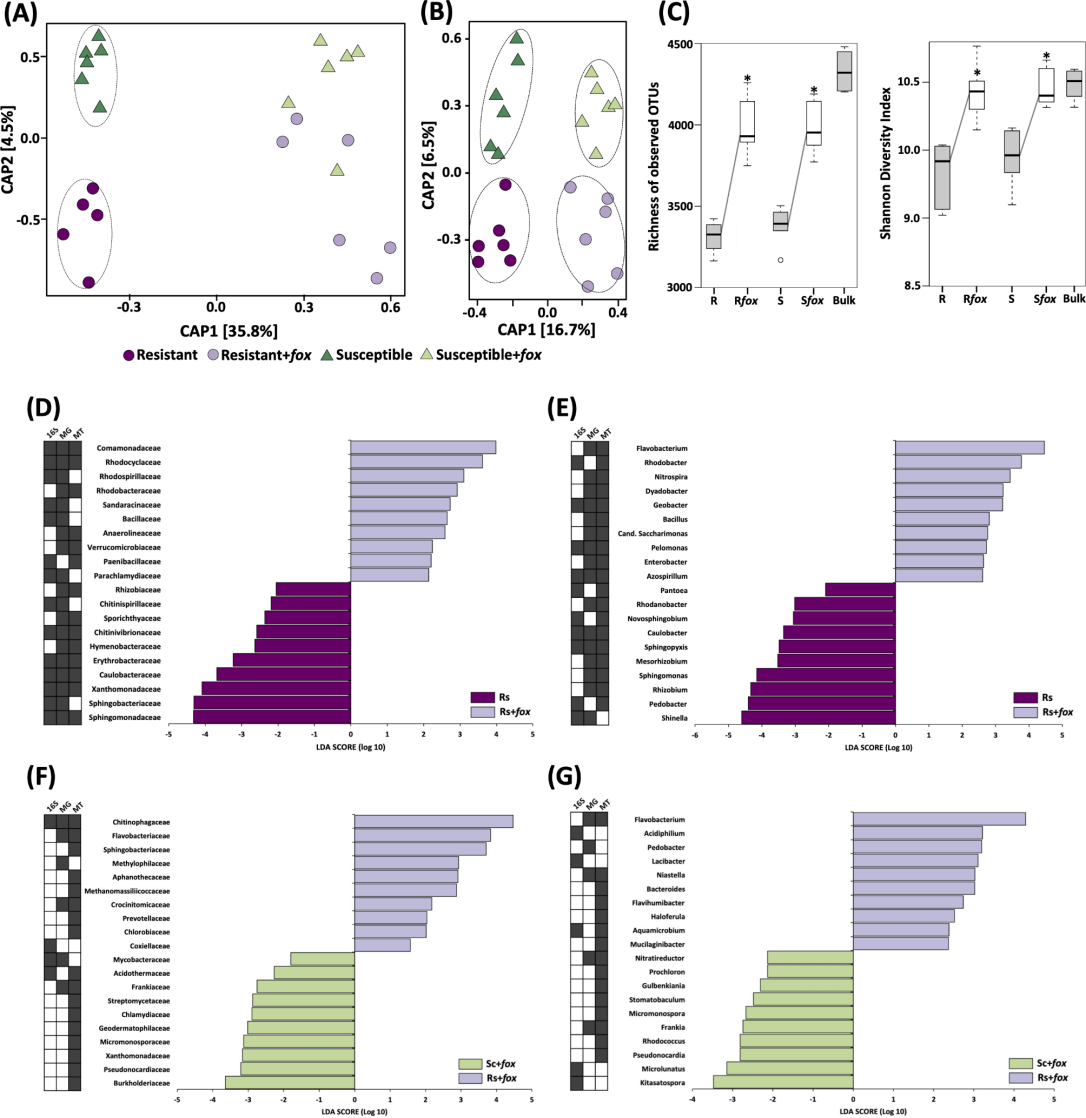
Structure, diversity and composition of the rhizosphere bacterial communities associated with two common bean cultivars non-inoculated or inoculated with *Fusarium oxysporum (Fox)*. Principal coordinate analsyis (CAP) comparing the communities structures in rhizosphere microbiome using **(A)** 16S and **(B)** metagenome data. Significant clusters (PERMANOVA, P < 0.05) are indicated by lines in the CAP graphs. **(C)** Diversity measurements in the rhizosphere microbiome. Asterisks in the diversity graphs indicate signifcant differences based on Tukey’s test (P < 0.05). Linear discriminant analysis (LDA) Effect Size (LEfSe) of microbial taxa enriched after *Fox* inoculation in the rhizosphere of the *fox*-resistant cultivar at **(D)** family and **(E)** genus level. Comparison of the microbial taxa enriched in the rhizosphere of *fox*-resistant and susceptible cultivar with *fox* inoculation at **(F)** family and **(G)** genus level. Filled squares at the left side of the graphs indicate the datasets were the differential taxa were found. R = *fox*-resistant cultivar; R*fox* = *fox*-resistant cultivar infected; S = susceptible cultivar; S*fox* – susceptible cultivar infected; Bulk = bulk soil

To understand the effect of the fungal root pathogen on the rhizosphere microbiome structure, we compared the samples based on the Constrained Analysis of Principal Coordinates (CAP), which revealed a distinct response of the bacterial community to pathogen invasion (Fig. 3A). The rhizosphere microbiome structure was different between *fox*-resistant and the susceptible cultivar in the non-inoculated treatment (*P* = 0.01), while after *fox* infection the community became different from the non-infected treatments, but with no significant difference between the cultivars based on 16S rRNA (Fig. 3A; Supplementary Table 2). Additionally, we also compared the community structure based on the metagenome data and that analysis revealed a distinct microbiome for the cultivar and treatment (Fig. 3B). Together, these results indicate that each common bean cultivar assembles a distinct rhizospheric community from the same soil microbial inoculum, which subsequently displays a different response to pathogen invasion.

To gain insights into the diversity parameters we compared the total number of observed OTUs and Shannon’s index of the communities retrieved from the three niches (Fig. 3C). All the indices revealed a significant increase in bacterial richness and diversity in the treatments with *fox* infection in the rhizosphere (Tukey’s HSD, *P* < 0.05). Also, the pathogen infection affected the proportion of generalists and specialists across the treatments (Supplementary Fig. 5). In general, pathogen infection increased the proportion of specialists in the rhizosphere of both common bean cultivars, with the *fox*-resistant cultivar presenting a higher proportion of specialists (from 4.5 to 11.7%, 67 specialists in total) than the susceptible cultivar (from 4.3 to 10.3%, 26 specialists in total). This result suggests a selection of specific microbial groups in the rhizosphere after pathogen infection.

Next, we compared the community composition using the three datasets at two taxonomic levels using LEfSe analysis. The results revealed a significant increase in the abundance of bacterial families after pathogen infection. Here, we highlight the top eight families that increased in at least two of the three datasets, namely Comamonadaceae, Rhodocyclaceae, Rhodospirillaceae, Sandaracinaceae, Bacillaceae, Anaerolineaceae, Verrucomicrobiaceae, and Paenibacillaceae (Fig. 3D). The same pattern was observed at the genus level, with a large number of genera with higher abundance after pathogen infection. The top 10 bacterial genera with higher abundance in the *fox-*resistant cultivar after infection were *Flavobacterium*, *Rhodobacter*, *Nitrospira*, *Dyadobacter*, *Geobacter*, *Bacillus*, *Candidatus Saccharimonas*, *Pelomonas*, and *Enterobacter* (Fig. 3E).

We further compared the effect of *fox* invasion between the *fox*-resistant and the susceptible cultivars. The results revealed several bacterial families and genera that responded differently to the pathogen. Compared to the susceptible cultivar, the *fox*-resistant cultivar presented an increased abundance of the families Chitinophagaceae, Flavobacteriaceae, Sphingobacteriaceae, Methylophilaceae, Aphanothecaceae, Methanomassiliicoccaceae, Crocinitomicaceae, Prevoletaceaea, and Chlorobiaceae (Fig. 3F). At a deeper taxonomic level, the top nine bacterial genera that presented higher abundance in the infected *fox*-resistant cultivar as compared with the susceptible cultivar were members of Bacteroidetes (*Flavobacterium*, *Pedobacter*, *Lacibacter*, *Niastella*, *Bacteroides*, *Flavihumibacter spp.*), Alphaproteobacteria (*Acidiphilium* and *Aquamicrobium*), and Verrucomicrobia (*Haloferula* spp.) (Fig. 3G).

In conclusion, we found that pathogen invasion enhanced the overall diversity (Fig. 3C) and abundance of specific microbial groups (Fig. 3D-G), such as members from Bacteroidetes, Alphaproteobacteria, and Verrucomicrobia in the rhizosphere microbiome and this response was distinct between the *fox*-resistant and susceptible cultivars.

#### Changes in rhizosphere microbiome functions of plants exposed to the pathogen

We subsequently examined if certain gene categories were enriched or depleted in the rhizosphere microbiome as compared to bulk soil and rhizosphere of non-infected and infected plants. To this end, we used 26 broad functional gene categories based on the COG database [72] (Supplementary Table 5). Principal Coordinate analysis based on the microbiome functional COG categories showed significant differences between the treatments (Supplementary Fig. 6A) (P < 0.05). Further analysis using diversity indices showed a significant decrease in functional diversity after pathogen infection (Supplementary Fig. 6B). Comparing with bulk soil, our rhizosphere samples presented an increase of genes affiliated to metabolism and transport of carbohydrates and inorganic ions, cell motility, biogenesis of cell wall and membrane, and signal transduction mechanisms (Supplementary Fig. 7A). Analyzing the response of the rhizosphere microbiome of the *fox*-resistant cultivar to the pathogen, our analysis showed an enrichment of genes related to defense mechanism, RNA processing and modification, signal transduction mechanisms, energy production and conversion (Supplementary Fig. 7B and 8). Comparing the responses between the two contrasting cultivars, the analysis showed that the resistant cultivar presented a higher abundance of genes related to defense mechanisms, nucleotide transport and metabolism, signal transduction mechanisms, biogenesis of cell wall membranes and replication, recombination and repair (Supplementary Fig. 9A and B). To better understand the effect of the pathogen invasion on the rhizosphere microbiome, we then compared the samples at a deeper functional level (COG) using LEfSe analysis. From the total of 4,631 COGs detected in all samples, 53 genes comprising 15 functions were overrepresented in the microbiome of the *fox*-resistant cultivar after pathogen infection (Supplementary Table 6). Comparing the response to pathogen infection between the two contrasting cultivars, we detected 26 COGs that were more present in the microbiome of the *fox*-resistant rhizosphere as compared to the susceptible cultivar (Supplementary Table 7).

**Fig. 4:**
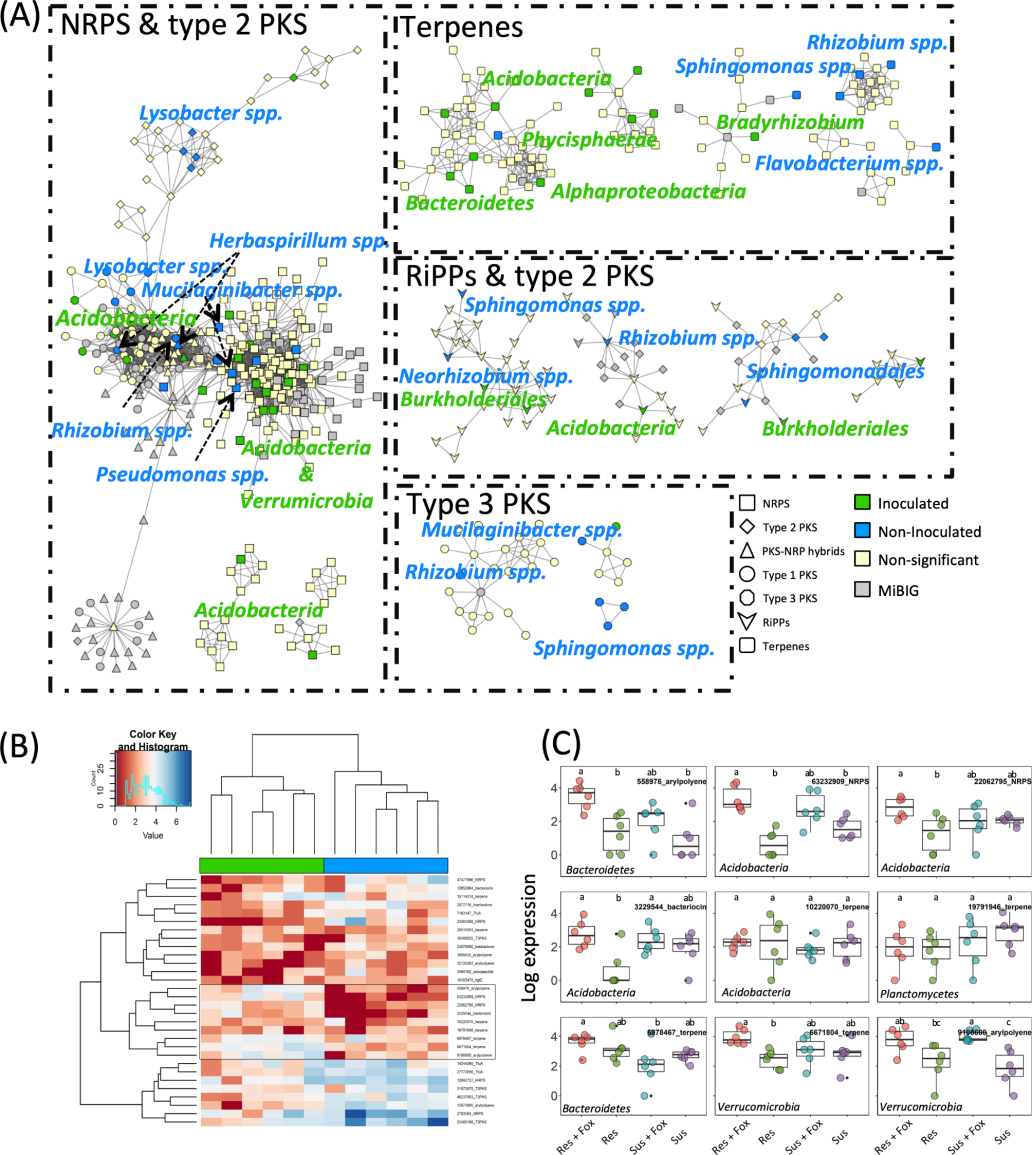
Diversity and distribution of biosynthetic gene clusters (BGC) in the rhizosphere microbiome of the *fox*-resistant common bean cultivar inoculated and non-inoculated with *Fusarium oxysporum*. **(A)** Sequence similarity network [constructed with BiG-SCAPE (Navarro-Muñoz et al., 2019), threshold: 0.4] of the different classes of BGCs detected in the rhizosphere microbiome. Taxonomic assignment and BGC class annotation of the nodes are shown. Nodes with fewer than three connections were removed. Node colors represent statistical significance (FDR < 0.05): Yellow nodes are non significant, green and blue nodes are significantly overrepresented in bean plants inoculated and non-inoculated with *F. oxysporum*. **(B)** Clustered heat map of the differentially expressed BGCs among the different treatments. **(C)** Subset of the 9 BGCs that are significantly overexpressed in the resistant cultivar inoculated with the pathogen

We then investigated which biosynthetic gene clusters (BGCs) were expressed during pathogen infection in the rhizosphere and/or significantly more abundant in both compartments. The prediction of BGCs using antiSMASH revealed a total of 862 BGCs associated with the biosynthesis of nonribosomal peptides, polyketides, terpenes, aryl polyenes, ribosomally synthesized and post-translationally modified peptides (RiPPs), phosphonates, phenazines, and siderophores (Fig. 4A, Supplementary Fig. 10 and Supplementary Table 8). Our analysis showed that the BGC structure presented differences between niches and treatments (P < 0.05)(Supplementary Fig. 11). In the rhizosphere, the *fox*-resistant and susceptible cultivars presented a significant overrepresentation of 131 and 48 BGC genes, respectively (Supplementary Fig. 10 and Supplementary Tables 9 and 10). Among the overrepresented BGCs in each of the treatments, 58 and 27 were more abundant when the pathogen was inoculated.

Remarkably, the metatranscriptome data showed that only nine and 12 BGCs were differently abundant in the *fox*-resistant and susceptible cultivars, respectively (Supplementary Tables 11 and 12). Of these, six and 10 BGCs were significantly overexpressed upon pathogen infection in the *fox*-resistant and susceptible cultivars, respectively (Fig. 4B and C). These results indicate that the rhizosphere microbiome of the *fox*-resistant cultivar is more responsive to the pathogen infection. The statistical analysis of the BGCs showed that most of the overexpressed clusters under pathogen infection belonged to the terpenes class (Fig. 4B and C). A further comparison of the increased (metagenome) and overexpressed (metatranscriptome) BGCs showed five statistically significant clusters belonging to terpenes, NRPS-like, NRPS, betalactone, and arylpolyene, which were affiliated to the bacterial groups Acidobacteria, Myxococcales, Burkholderiales, and Flavobacteriales (Fig. 4C). Interestingly, the BGC arylpolyene assigned to Flavobacteriales had almost no reads in the metagenome but was highly expressed (logFold = 3.86) in the metatranscriptome after *fox* infection. It is worth noting that members of the Flavobacteriales order were among the most responsive taxa under pathogen infection.

### Microbiome response to pathogen invasion in the endosphere

#### Changes in endosphere bacterial community composition

**Fig. 5:**
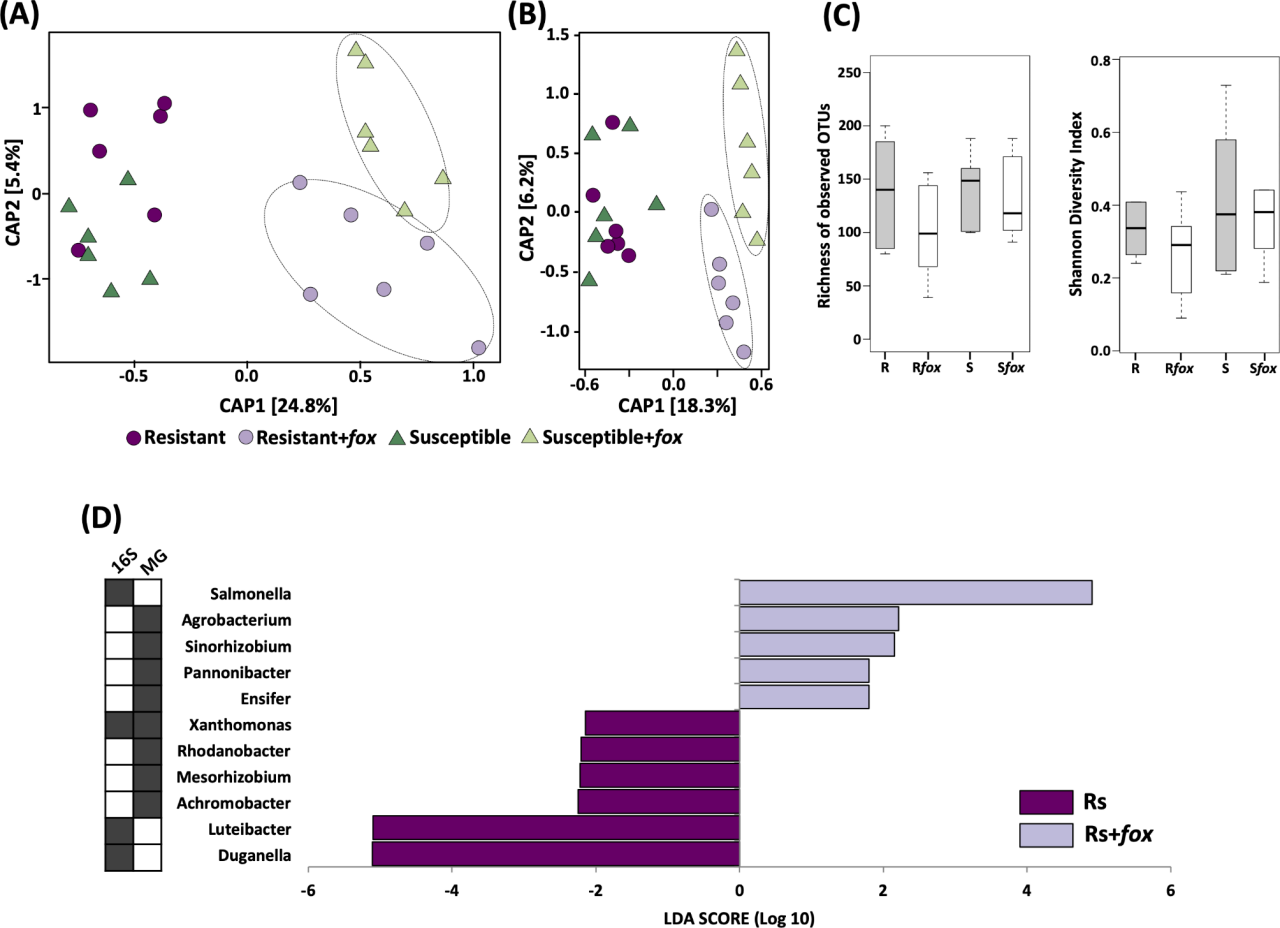
Structure, diversity and composition of the endosphere bacterial community from two common bean cultivars non-inoculated or inoculated with *Fusarium oxysporum (fox)*. Principal coordinate analysis (CAP) comparing the endophytic community using **(A)** 16S rRNA and **(B)** metagenome data. Significant clusters (PERMANOVA, P < 0.05) are indicated by lines in the CAP graphs. **(C)** Diversity measurements for the endophytic community. Asterisks in the diversity graphs indicate signifcant differences based on Tukey’s test (P < 0.05). **(D)** Linear discriminant analysis (LDA) Effect Size (LEfSe) of microbial taxa enriched after *fox* inoculation in the endophytic community of the *fox*-resistant cultivar at genus level. Filled squares at the left side of the graphs indicate the datasets were the differential taxa were found. R = *fox*-resistant cultivar; R*fox* = *fox*-resistant cultivar infected; S = susceptible cultivar; S*fox* – susceptible cultivar infected; Bulk = bulk soil

For the endosphere, the microbiome response presented a reverse pattern when compared with the rhizosphere, where the endosphere microbiome of the susceptible cultivar was more altered by the root pathogen. The communities were similar between cultivars in the non-inoculated treatment and became distinct (*P* = 0.002) after *fox* inoculation, for both 16S rRNA and metagenome data (Fig. 5A and B; Supplementary Table 2). For the diversity of the endophytic community, there was no difference between the treatments (Tukey’s HSD, *P* > 0.05) (Fig. 5C). The analysis of niche occupancy revealed that the proportion of specialists in the endosphere of the *fox*-resistant cultivar decreased after the pathogen infection (from 24.1 to 7.6%) and increased in the susceptible cultivar (average 16.5%) (Supplementary Fig. 12). Taken together, these results suggest a prompt response of the endophytic microbial community in the presence of the pathogen, with the susceptible cultivar being more responsive.

To assess the endophytic response of the *fox*-resistant cultivar to pathogen invasion, we compared the community composition at two taxonomic levels. The LEfSe analysis did not point to any specific family in the endosphere of the *fox*-resistant cultivar that increased in abundance after pathogen infection. At the genus level, however, the analysis showed that *Agrobacterium*, *Sinorhizobium*, *Salmonella*, *Ensifer*, and *Pannonibacter* increased after *fox* infection in the *fox*-resistant endophytic root compartment (Fig. 5D). A further analysis comparing the effects of *fox* infection between the *fox*-resistant cultivar and the susceptible cultivar showed small differences, with an increase in the abundance of some families and genera in the susceptible cultivar. Only the genus *Candidatus Entotheonella* was more abundant in the infected *fox*-resistant cultivar in comparison with the susceptible cultivar. This genus could suppress the pathogen by producing antibiotics and polyketides [101].

#### Changes in endosphere microbiome functions of plants exposed to the pathogen

The functional analysis of the endosphere microbiome is based on metagenome analyses only. In contrast to the rhizosphere, we could not conduct a metatranscriptome analysis because of technical limitations due to the substantial RNA contamination from the plant tissues. The COG analysis revealed that the pathogen affected the functional structure of both *fox*-resistant and susceptible cultivars (Supplementary Fig. 13). Comparing the samples at a deeper functional COG level using LEfSe analysis, we identified 35 genes comprising 11 functions that were enriched in the endosphere of the *fox*-resistant cultivar upon pathogen infection (Supplementary Table 13). Comparing the two contrasting cultivars, we detected two COGs that were more abundant in the *fox*-resistant endosphere, both classified as transposases (Supplementary Table 14).

Lastly, our results showed that pathogen infection had a strong effect on the composition of the BGCs (P < 0.05) (Supplementary Figs. 14–16). We then investigated which BGCs were up-regulated during pathogen infection in the endosphere and we found that the susceptible cultivar was more responsive compared to the *fox*-resistant. In the resistant, only 12 out of 50 differential BGCs were more abundant when the plant was challenged with the pathogen (Supplementary Tables 15 and 16), while in the susceptible cultivar 27 out of 64 increased in abundance upon pathogen infection. Interestingly, terpenes were also a major class in the endosphere of the resistant cultivar but were more abundant in the non-inoculated treatment.

## Discussion

In this study, we showed that root pathogenic *F. oxysporum* (*fox*) had a significant impact on the taxonomic and functional diversity of the rhizosphere and endosphere microbiome of common bean cultivars with distinct levels of *fox*-resistance. First, we observed marked differences in the structure and composition of the microbial communities associated with each niche, i.e. bulk soil, rhizosphere, and endosphere. Remarkably, Proteobacteria exceeded 95% of the endosphere microbiome, with 99% of these sequences belonging to the *Rhizobium* genus, a well-known endosymbiotic nitrogen-fixing microbe associated with roots of leguminous species. Interestingly, the microbiome assembly in each niche followed distinct patterns, with the rhizosphere samples being dominated by niche-based mechanisms, while bulk soil and endosphere followed a neutral process. This result confirms and extends previous results that the rhizosphere microbiome is influenced more by selection processes associated with biotic and abiotic factors in this niche [73]. Indeed, we showed that pathogen infection led to significant changes in the rhizosphere community composition and structure, extending results from previous studies on banana [74], barley [75], citrus [76], cotton [77], and sugar beet [5, 13]. We also observed that the rhizosphere community responded to pathogen invasion by enhancing diversity, community complexity (i.e., number of interactions in the network), and a higher proportion of specialists. Enhanced microbial diversity together with higher community complexity could diminish pathogen invasion success due to a more efficient competition for resources and niche occupancy [78, 79]. Although both common bean cultivars showed an enhanced microbial diversity upon pathogen invasion, we found a higher number of specialists and more complexity in the *fox*-resistant cultivar. Previous studies have demonstrated that specialists have a narrow niche but the highest fitness in that niche [80, 81]. Also, specialists are more responsive to environmental disturbances [82, 83], such as pathogen invasion. *Paenibacillus*, *Saccharibacteria*, Chitinophagaceae, and *Flavobacterium* were found as specialists in the rhizosphere of the *fox*-resistant cultivar. Although several of these genera have been previously reported for their antagonistic activities toward pathogenic *Fusarium* species of different crops [84, 85], future experiments will be needed to validate this assumption.

Also, the mechanisms underlying the observed microbiome changes are to be elucidated. The *fox*-resistance of the resistant cultivar is genetically and physiologically based, where the pathogen invasion is restricted by vascular occlusion, tyloses, deposition of additional wall layers, and infusion of phenols and other metabolites [27]. This genetic change can alter plant exudation patterns and the assembly of the rhizosphere community, differentiating the microbiome assembly between the cultivars with distinct levels of resistance to *fox* [6, 28, 29]. Those microbiome members that are differentially enriched in the microbiome of the *fox*-resistant cultivar in absence of the pathogen may have complementary protective activities to the intrinsic genetic *fox*-resistance. In the current study, we showed that *fox* infection also had a significant impact on the microbial communities of the rhizosphere and endosphere of these cultivars. These microbiome shifts can be caused directly by the pathogen itself or indirectly via plant physiological changes induced by the pathogen. The latter mechanism has also been referred to as the ‘cry for help’ [86], where plants under siege secrete specific exudates or signaling compounds that recruit and or activate specific members of the root microbiome for protection against subsequent infections. Liu et al. [87] showed that local root infection by *F. oxysporum* in cucumber altered the concentration of 89 mostly primary metabolites in exudates, which correlated with root colonization by beneficial *Bacillus amyloliquefaciens*. Whether the changes we found in the community composition in the *fox*-resistant cultivar under the pathogen infection are the results of the induced excretion of antimicrobial compounds by the infected roots remains to be investigated.

Most studies on plant microbiome have focused more on microbial diversity rather than on gene function [88]. Microbes living on and in plant roots may induce known and yet unknown biosynthetic pathways in plants leading to alterations in the plant chemistry [89]. On the other hand, changes in plant metabolomics may affect the functional profile of the associated microbiome. Thus, we assessed the effect of the pathogen infection on the functional profiles of the microbiome. A common strategy used by microbes against other competitors includes limiting resources and producing antimicrobial compounds [90]. Interestingly, the *fox*-resistant cultivar presented an enrichment of sequences affiliated to ‘defense mechanisms’ after pathogen infection. The increase of sequences affiliated to this category could reflect the more diverse and dynamic the community becomes after the pathogen infection (based on niche occupancy and network analysis). Also, there is an increase of genes belonging to the pathway classified as ‘signal transduction mechanism’. This pathway can act to amplify the cellular response to an external signal, which could lead to a prompt response of the community towards the pathogen infection. Carrión et al. [7] found an enrichment of genes affiliated to signal transduction mechanisms in the endophytic community of sugar beet grown in suppressive soils in the presence of the pathogen *Rhizoctonia solani.* They also noted that specific bacterial families were associated with this enrichment, namely Chitinophagaceae, Flavobacteriaceae, and Pseudomonadaceae, groups that also increased in abundance in the rhizosphere and endosphere of the infected *fox*-resistant cultivar in our experiment. Later, the BGC analysis revealed several clusters enriched in both the rhizosphere and endosphere in presence of the pathogen. An important means of microbial protection are secondary metabolites, which are a very broad group of compounds or peptides with a wide range of biological activities, e.g., antimicrobial or iron chelation [91, 92]. Our analysis obtained five candidate BGCs, with terpenes as the most representative BGC class in both rhizosphere and endosphere. Although terpenes have mostly been isolated from plants and fungi, they are also widely distributed in bacteria [93]. It has been shown that the biosynthesis of terpenes by plants [94, 95] and bacteria [96] suppress *fox* infection. Thus, the high abundance of terpenes in the rhizosphere microbiome could indicate its role in the suppression of *fox* infection in the resistant bean cultivar. Interestingly, the BGC arylpolyene was highly expressed after *fox* invasion and a previous report has shown its function in the control of banana fusarium wilt [96]. Interestingly, the *fox*-resistant cultivar presented an increased expression of arylpolyene genes, which were affiliated to Flavobacteriaceae family, a group of bacteria that was the most responsive to pathogen infection in this cultivar. The genus *Flavobacterium* is reported to suppress *fox* in several plant species [84, 97]. It’s worth noting that the rhizosphere of the *fox*-resistant cultivar was more responsive to the pathogen infection. On the other hand, the endosphere of the susceptible cultivar presented more overrepresented BGCs, suggesting that this cultivar is more affected by the pathogen infection, revealing less efficiency of the susceptible rhizosphere microbiome to protect the plant against the pathogen infection. Together, our analysis of the functional profile indicates a pathogen-induced activation of disease-suppressive functions in the rhizosphere and endosphere of the *fox*-resistant cultivar, suggesting that breeding for *fox* resistance in common bean may have co-selected for unknown plant traits that reinforce microbiome-assisted plant defense.

## Conclusions

Our multi-‘omics approach allowed us to identify the most responsive bacterial groups in the common bean rhizosphere and endosphere to invasion by the fungal root pathogen *Fusarium oxysporum*. We found that the genera *Flavobacterium*, *Dyadobacter*, *Bacillus*, *Pedobacter, Pseudomonas*, and *Paenibacillus* were enriched in the rhizosphere and endosphere of the *fox*-resistant cultivar under siege. Interestingly, the genus *Flavobacterium* showed up as the most responsive species, increasing in abundance and identified as keystone species and a specialist group. These responsive species may display different mechanisms in disease suppression, including competition for nutrition and ecological niches, production of antibiotics, and induction of plant systemic resistance [13, 98]. Our metatranscriptome analysis showed that the root microbiome of the *fox*-resistant cultivar was more responsive to the pathogen invasion, with a higher expression of biosynthetic gene clusters classified as terpenes, NRPS-like, NRPS, betalactone, and arylpolyene. Whether the enriched members and traits of the root microbiome reinforce the resistance of the *fox*-resistant cultivar or if the changes in the microbiome are a consequence of the fungal invasion remains to be investigated. For this, a comprehensive study would involve the isolation of antagonistic microbial groups, as pointed by metagenome approach and selected from the rhizosphere of the *fox*-resistant cultivar. This would be combined with the use of site-directed mutagenesis to identify and confirm specific microbial antagonistic traits responsible for the soil borne pathogen antagonism. Additionally, a metabolomic approach would be instrumental for identifying plant compounds responsible for both microbial recruitment and/or pathogen antagonism. Lastly, we emphasize that next-generation sequencing coupled with a community ecology approach is pivotal to help disentangle the link between plant defense and root-associated microbial communities.

### Electronic supplementary material

Below is the link to the electronic supplementary material.

**Supplementary Figure 1**. (A) Confirmation of plant infection by isolation of *Fusariumoxysporum* from root fragments on PDA medium. (B) Plant infection symptoms of *Fusariumoxysporum* infection. **Supplementary Figure 2**. Overall composition of bacterial phyla identified in bulk soil, rhizosphere and endosphere using three datasets (16S rRNA, metagenome and metatranscriptome). **Supplementary Figure 3**. Linear discriminant analysis (LDA) Effect Size (LEfSe) analysis of bacterial phyla present in bulk soil, rhizosphere and endosphere of common bean for (A) 16S rRNA, (B) metagenome, and (C) metatranscriptome. Red bars refer to significant abundant taxa in bulk soil, while purple refers to rhizosphere and green to endosphere. **Supplementary Figure 4**. Akaike Information Criterion (AIC) weight values for six rank abundance distribution models used in this work. The AIC weight varies from 0 to 1, being the highest value the best-fit model. The color scale was used for a better visualization, where green indicates the best model. **Supplementary Figure 5**. Multinomial species classification method (CLAM) for the niche occupancy test for the rhizosphere microbiome. The niche occupancy was evaluated in pairwise comparison between the treatments. The percentage of specialists is indicated in the graphs. R = *fox*-resistant cultivar; Rfox = *fox*-resistant cultivar infected; S = susceptible cultivar; Sfox = susceptible cultivar infected; Bulk = bulk soil. **Supplementary Figure 6**. Structure and diversity of rhizopshere and bulk soil functional profile (based on COG) from two common bean cultivars non-inoculated or inoculated with *Fusarium oxysporum* (*fox*). Principal component analsyis (PCA) comparing the functional profile structure in the rhizosphere microbiome using (A) metagenome and (C) metatranscriptome. Diversity measurements of the rhizopshere functional profile using (B) metagenome and (D) metatranscriptome. R = *fox*-resistant cultivar; Rfox = *fox*-resistant cultivar infected; S = susceptible cultivar; Sfox = susceptible cultivar infected; Bulk = bulk soil. **Supplementary Figure 7**. Scatter-plot showing the differential abundance of sequences affiliated to bacterial functions between bulk soil and rhizosphere of common bean. The sequences were affiliated to functional categories based on COG database using (A) metagenome and (B) metatranscriptome datasets. Asterisks indicate enriched categories in the rhizosphere common to both datasets. P-values were calculated using Welch’s t-test with Benjamini-Hochberg correction (P < 0.05). A list with all the COG categories is shown in the right side of the figure. **Supplementary Figure 8**. Scatter-plot showing the differential abundance of sequences affiliated to bacterial functions in the *fox*-resistant cultivar after *fox* inoculation. The sequences were affiliated to functional categories based on COG database using (A) metagenome and (B) metatranscriptome datasets. P-values were calculated using Welch’s t-test with Benjamini-Hochberg correction (P < 0.05). A list with all the COG categories is shown in the right side of the figure. **Supplementary Figure 9**. Scatter-plot showing the differential abundance of sequences affiliated to bacterial functions comparing the *fox*-resistant cultivar with the susceptible after *fox* inoculation. The sequences were affiliated to functional categories based on COG database using (A) metagenome and (B) metatranscriptome datasets. P-values were calculated using Welch’s t-test with Benjamini-Hochberg correction (P < 0.05). A list with all the COG categories is shown in the right side of the figure. **Supplementary Figure 10**. Diversity and distribution of biosynthetic gene clusters in the rhizosphere microbiome of the susceptible common bean cultivar, inoculated and non-inoculated with *Fusarium oxysporum*. Sequence similarity network (constructed with BiG-SCAPE, threshold: 0.4) of the different classes of BGCs detected in the rhizosphere microbiome. Taxonomic assignment and BGC class annotation of the nodes are shown. Nodes with fewer than three connections were removed. Node colors represent statistical significance (FDR < 0.05): Yellow nodes are nonsignificant, green and blue nodes are significantly overrepresented in bean plants inoculated and non-inoculated with *fox*. **Supplementary Figure 11**. Structure of the rhizopshere and bulk soil functional profile (based on Biosynthetic Gene Clusters) from two common bean cultivars non-inoculated or inoculated with *Fusarium oxysporum *(*fox*). Principal coordinate analsyis (PCoA) comparing the functional profile structure in the rhizosphere of the two common bean cultivars after *fox* inoculation using (A) metagenome and (B) metatranscriptome. **Supplementary Figure 12**. Multinomial species classification method (CLAM) for the niche occupancy test for the endosphere microbiome. The niche occupancy was evaluated in pairwise comparison between the treatments. The percentage of specialists is indicated in the graphs. R = *fox*-resistant cultivar; Rfox = *fox*-resistant cultivar infected; S = susceptible cultivar; Sfox = susceptible cultivar inoculated. **Supplementary Figure 13**. Structure and diversity of endosphere functional profile (based on COG) from two common bean cultivars non-inoculated or inoculated with *Fusarium oxysporum * (*fox*). (A) Principal component analsyis (PCA) comparing the functional profile structures using metagenome data. (B) Diversity measurements of the endosphere functional profile using metagenome. R = *fox*-resistant cultivar; Rfox = *fox*-resistant cultivar infected; S = susceptible cultivar; Sfox = susceptible cultivar infected. **Supplementary Figure 14**. Principal coordinate analsyis (PCoA) comparing the endosphere functional profile structure (based on Biosynthetic Gene Clusters) in the rhizosphere of the two common bean cultivars non-inoculated or inoculated with *Fusarium oxysporum* (*fox*) using metagenome data. **Supplementary Figure 15**. Diversity and distribution of biosynthetic gene clusters in the endosphere microbiome of the resistant common bean cultivar, non-inoculated or inoculated with *Fusarium oxysporum* (*fox*). Sequence similarity network (constructed with BiG-SCAPE, threshold: 0.4) of the different classes of BGCs detected in the rhizosphere microbiome. Taxonomic assignment and BGC class annotation of the nodes are shown. Nodes with fewer than three connections were removed. Node colors represent statistical significance (FDR < 0.05): Yellow nodes are nonsignificant, green and blue nodes are significantly overrepresented in bean plants inoculated and non-inoculated with *fox*. **Supplementary Figure 16**. Diversity and distribution of biosynthetic gene clusters in the endosphere microbiome of the susceptible common bean cultivar, non-inoculated or inoculated with *Fusarium oxysporum* (*fox*). Sequence similarity network (constructed with BiG-SCAPE, threshold: 0.4) of the different classes of BGCs detected in the rhizosphere microbiome. Taxonomic assignment and BGC class annotation of the nodes are shown. Nodes with fewer than three connections were removed. Node colors represent statistical significance (FDR < 0.05): Yellow nodes are nonsignificant, green and blue nodes are significantly overrepresented in bean plants inoculated and non-inoculated with *fox*.

**Supplementary Table 1**. Soil analysis of Vredepeel field. **Supplemantary Table 2**. Results of PERMANOVA analysis of the Bray-Curtis dissimilarities for microbial community structure based on 16S rRNA gene at OTU level. Bold face indicates statistical significance (P < 0.05). P-values are based in 9999 permutations. **Supplementary Table 3**. Number of correlations per phylum. Showing the top five phyla with most correlation per treatment. **Supplementary Table 4**. Top five OTUs with more number of correlations (that is, degree) and betweeness centrality for each treatment. **Supplementary Table 5**. List of 26 COG categories. **Supplementary Table 6**. Differential expression of COG genes in rhizosphere comparing the non-inoculated fox-resistant cultivar (Rs) with the pathogen infection (Rs+fox), based on LDA Effect Size analysis. **Supplementary Table 7**. Differential expression of COG genes comparing the *fox*-resistant cultivar infected (Rs+fox) with the susceptible cultivar infected (Sc+fox), based on LDA Effect Size analysis. **Supplementary Table 8**. Analysis of the Biosynthetic Gene Clusters of the rhizosphere samples from common bean cultivars. **Supplementary Table 9**. Analysis of the abundance of Biosynthetic Gene Clusters in the rhizosphere comparing the non-inoculated fox-resistant cultivar (Rs) with the pathogen infection (Rs+fox). **Supplementary Table 10**. Analysis of the abundance of Biosynthetic Gene Clusters in the rhizosphere comparing the non-inoculated susceptible cultivar (Sc) with the pathogen infection (Sc+fox). **Supplementary Table 11**. Analysis of the abundance of Biosynthetic Gene Clusters in the rhizosphere of the fox-resistant cultivar (Rs). **Supplementary Table 12**. Analysis of the abundance of Biosynthetic Gene Clusters in the rhizosphere of the susceptible cultivar (Sc). **Supplementary Table 13**. Differential expression of COG genes in the endosphere of common bean comparing the non-inoculated *fox*-resistant cultivar (Rs) with the pathogen infection (Rs+fox), based on LDA Effect Size analysis. **Supplementary Table 14**. Differential expression of COG genes in the endosphere of common bean comparing the infected *fox*-resistant cultivar (Rs+*fox*) with the infected susceptible (Sc+*fox*), based on LDA Effect Size analysis. **Supplementary Table 15**. Analysis of the abundance of Biosynthetic Gene Clusters in the endosphere comparing the non-inoculated fox-resistant cultivar (Rs) with the pathogen infection (Rs+fox). **Supplementary Table 16**. Analysis of the abundance of Biosynthetic Gene Clusters in the endosphere of the susceptible common bean cultivar comparing the non-inoculated (Sc) with the pathogen-inoculated (Sc+fox) treatment.

## Data Availability

The datasets supporting the results and conclusions of this article were deposited in the NCBI Sequence Read Archive dataset under the accession numbers PRJNA904225 (16S rRNA), PRJNA904562 (metagenome), and PRJNA904281 (metatranscriptome). The codes used in the analysis can be found at Zenodo (10.5281/zenodo.7447085). All other data are contained within the main manuscript and supplementary material.
