# Supplementary material for: Impact of the fungal pathogen Fusarium oxysporum on the taxonomic and functional diversity of the common bean root microbiome
Source: Environ Microbiome. 2023 Aug 3;18:68. doi: 10.1186/s40793-023-00524-7 (PMC10401788; doi:10.1186/s40793-023-00524-7)
Supplement: Supplementary file 1 — Supplementary Figure 1. (A) Confirmation of plant infection by isolation of Fusariumoxysporum from root fragments on PDA medium. (B) Plant infection symptoms of Fusariumoxysporum infection. Supplementary Figure 2. Overall composition of bacterial phyla identified in bulk soil, rhizosphere and endosphere using three datasets (16S rRNA, metagenome and metatranscriptome). Supplementary Figure 3. Linear discriminant analysis (LDA) Effect Size (LEfSe) analysis of bacterial phyla present in bulk soil, rhizosphere and endosphere of common bean for (A) 16S rRNA, (B) metagenome, and (C) metatranscriptome. Red bars refer to significant abundant taxa in bulk soil, while purple refers to rhizosphere and green to endosphere. Supplementary Figure 4. Akaike Information Criterion (AIC) weight values for six rank abundance distribution models used in this work. The AIC weight varies from 0 to 1, being the highest value the best-fit model. The color scale was used for a better visualization, where green indicates the best model. Supplementary Figure 5. Multinomial species classification method (CLAM) for the niche occupancy test for the rhizosphere microbiome. The niche occupancy was evaluated in pairwise comparison between the treatments. The percentage of specialists is indicated in the graphs. R = fox-resistant cultivar; Rfox = fox-resistant cultivar infected; S = susceptible cultivar; Sfox = susceptible cultivar infected; Bulk = bulk soil. Supplementary Figure 6. Structure and diversity of rhizopshere and bulk soil functional profile (based on COG) from two common bean cultivars non-inoculated or inoculated with Fusarium oxysporum (fox). Principal component analsyis (PCA) comparing the functional profile structure in the rhizosphere microbiome using (A) metagenome and (C) metatranscriptome. Diversity measurements of the rhizopshere functional profile using (B) metagenome and (D) metatranscriptome. R = fox-resistant cultivar; Rfox = fox-resistant cultivar infected; S = susceptible [file 40793_2023_524_MOESM1_ESM.docx]

**SUPPLEMENTARY FIGURES**

**Impact of the fungal pathogen *Fusarium* *oxysporum* on the taxonomic and functional diversity of the common bean root microbiome**

Lucas William Mendes^1,2^*, Jos M Raaijmakers^2,3^, Mattias de Hollander^2^, Edis Sepo^3^, Ruth Gómez Expósito^2^, Alisson Fernando Chiorato^5^, Rodrigo Mendes^5^, Siu Mui Tsai^1^, Victor J Carrión^2,3,6*^

^1^Cell and Molecular Biology Laboratory, Center for Nuclear Energy in Agriculture CENA, University of Sao Paulo USP, 13416-000, Piracicaba, SP, Brazil; ^2^Departament of Microbial Ecology, Netherlands Institute of Ecology NIOO-KNAW, 6708 PB, Wageningen, The Netherlands; ^3^Institute of Biology, Leiden University, Leiden, the Netherlands; ^4^Instituto Agronômico IAC, Centro de Análises e Pesquisa Tecnológica do Agronegócio dos Grãos e Fibras, 130001-970, Campinas, Brazil; ^5^Laboratory of Environmental Microbiology, Embrapa Environment, 18020-000, Jaguariuna, Brazil; ^6^Departamento de Microbiología, Instituto de Hortofruticultura Subtropical y Mediterránea ‘La Mayora’, Universidad de Málaga-Consejo Superior de Investigaciones Científicas (IHSM-UMA-CSIC), Universidad de Málaga, Málaga, Spain

Supplementary Figures 1 to 16

**Supplementary Figure 1. (A)** Confirmation of plant infection by isolation of *Fusarium oxysporum* from root fragments on PDA medium. **(B)** Plant infection symptoms of *Fusarium* *oxysporum* infection.

**Supplementary Figure 2.** Overall composition of bacterial phyla identified in bulk soil, rhizosphere and endosphere using three datasets (16S rRNA, metagenome and metatranscriptome).

**Supplementary Figure 3.** Linear discriminant analysis (LDA) Effect Size (LEfSe) analysis of bacterial phyla present in bulk soil, rhizosphere and endosphere of common bean for **(A)** 16S rRNA, **(B)** metagenome and **(C)** metatranscriptome. Red bars refer to significant abundant taxa in bulk soil, while purple refers to rhizosphere and green to endosphere.


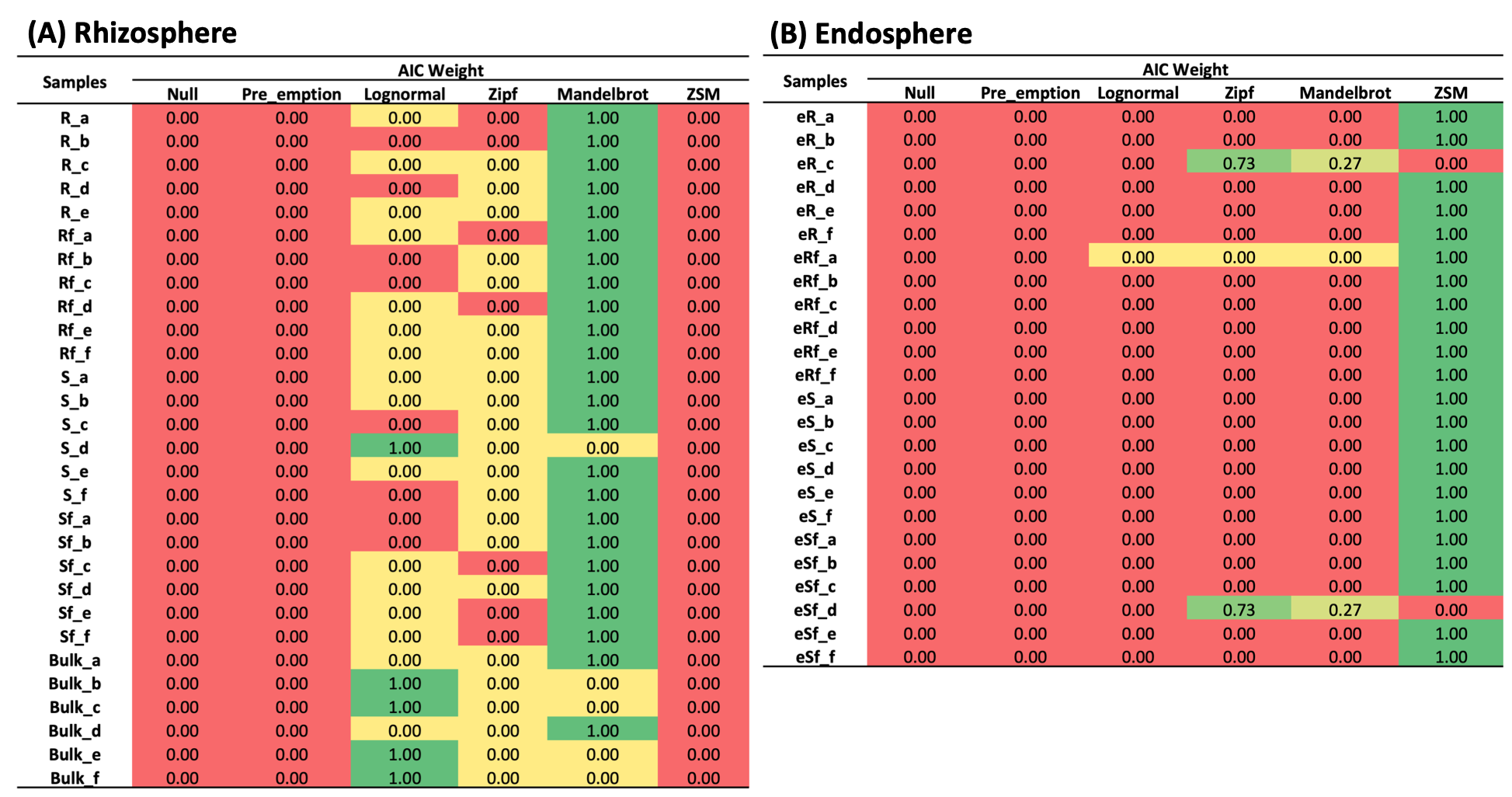


**Supplementary Figure 4.** Akaike Information Criterion (AIC) weight values for six rank abundance distribution models used in this work. The AIC weight varies from 0 to 1, being the highest value the best-fit model. The color scale was used for a better visualization, where green indicates the best model.

**Supplementary Figure 5.** Multinomial species classification method (CLAM) for the niche occupancy test for the rhizosphere microbiome. The niche occupancy was evaluated in pairwise comparison between the treatments. The percentage of specialists is indicated in the graphs. R = *fox*-resistant cultivar; R*fox* = *fox*-resistant cultivar infected; S = susceptible cultivar; S*fox* – susceptible cultivar infected; Bulk = bulk soil.


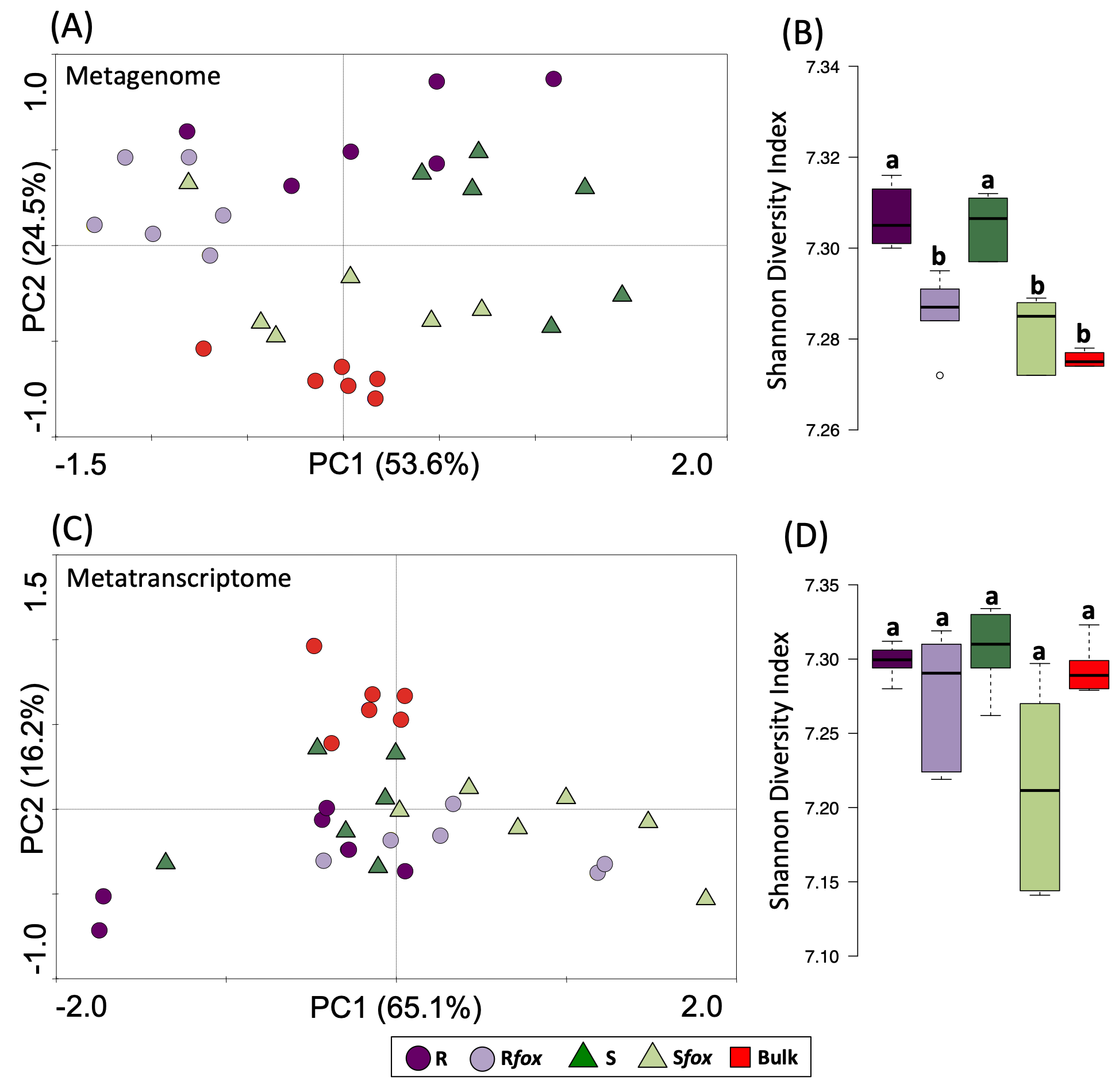


**Supplementary Figure 6.** Structure and diversity of rhizopshere and bulk soil functional profile (based on COG) from two common bean cultivars non-inoculated or inoculated with *Fusarium* *oxysporum (fox)*. Principal component analsyis (PCA) comparing the functional profile structure in the rhizosphere microbiome using **(A)** metagenome and **(C)** metatranscriptome. Diversity measurements of the rhizopshere functional profile using **(B)** metagenome and **(D)** metatranscriptome. R = *fox*-resistant cultivar; R*fox* = *fox*-resistant cultivar infected; S = susceptible cultivar; S*fox* – susceptible cultivar infected; Bulk = bulk soil.

**
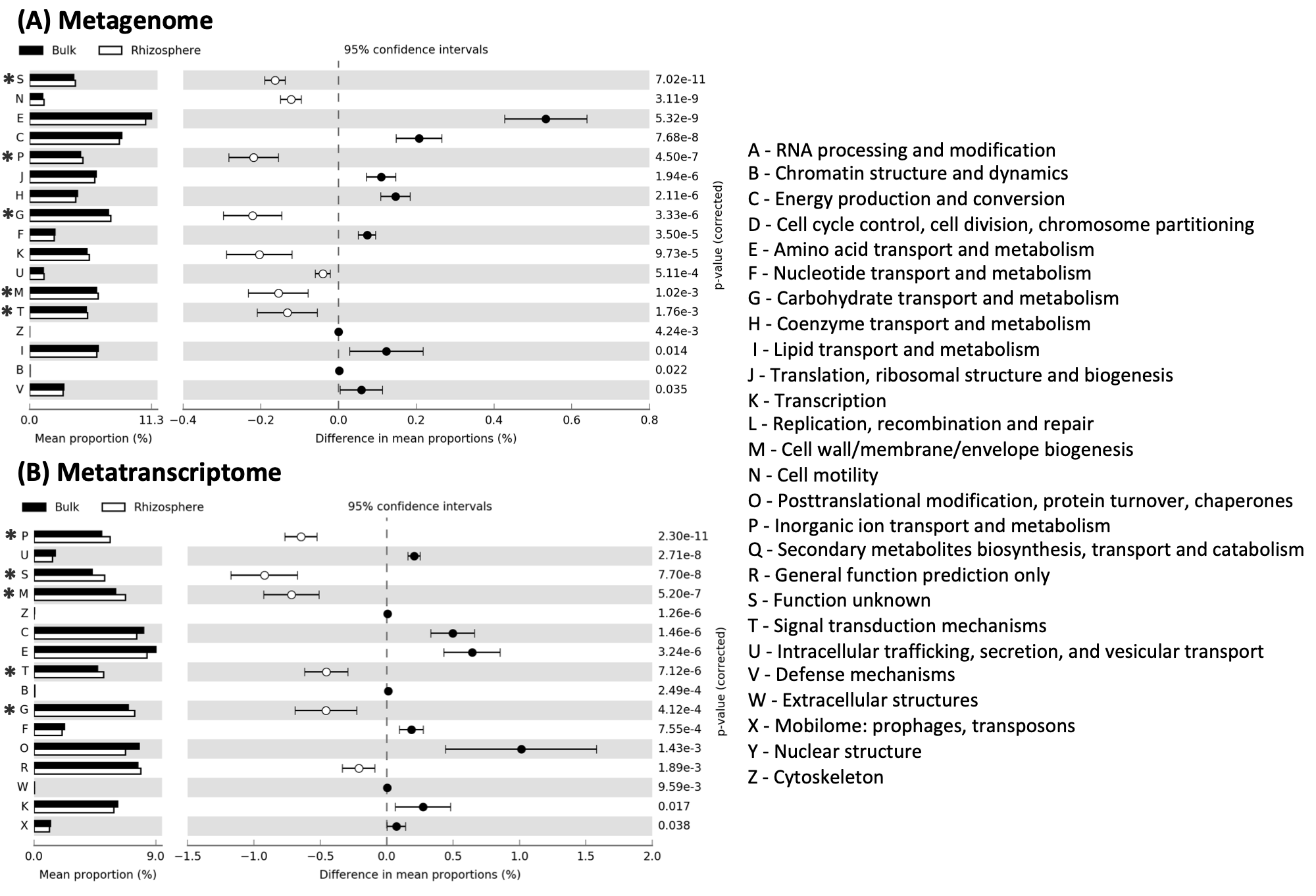
**

**Supplementary Figure 7.** Scatter-plot showing the differential abundance of sequences affiliated to bacterial functions between bulk soil and rhizosphere of common bean. The sequences were affiliated to functional categories based on COG database using **(A)** metagenome and **(B)** metatranscriptome datasets. Asterisks indicate enriched categories in the rhizosphere common to both datasets. P-values were calculated using Welch’s t-test with Benjamini-Hochberg correction (P < 0.05). A list with all the COG categories is shown in the right side of the figure.

**
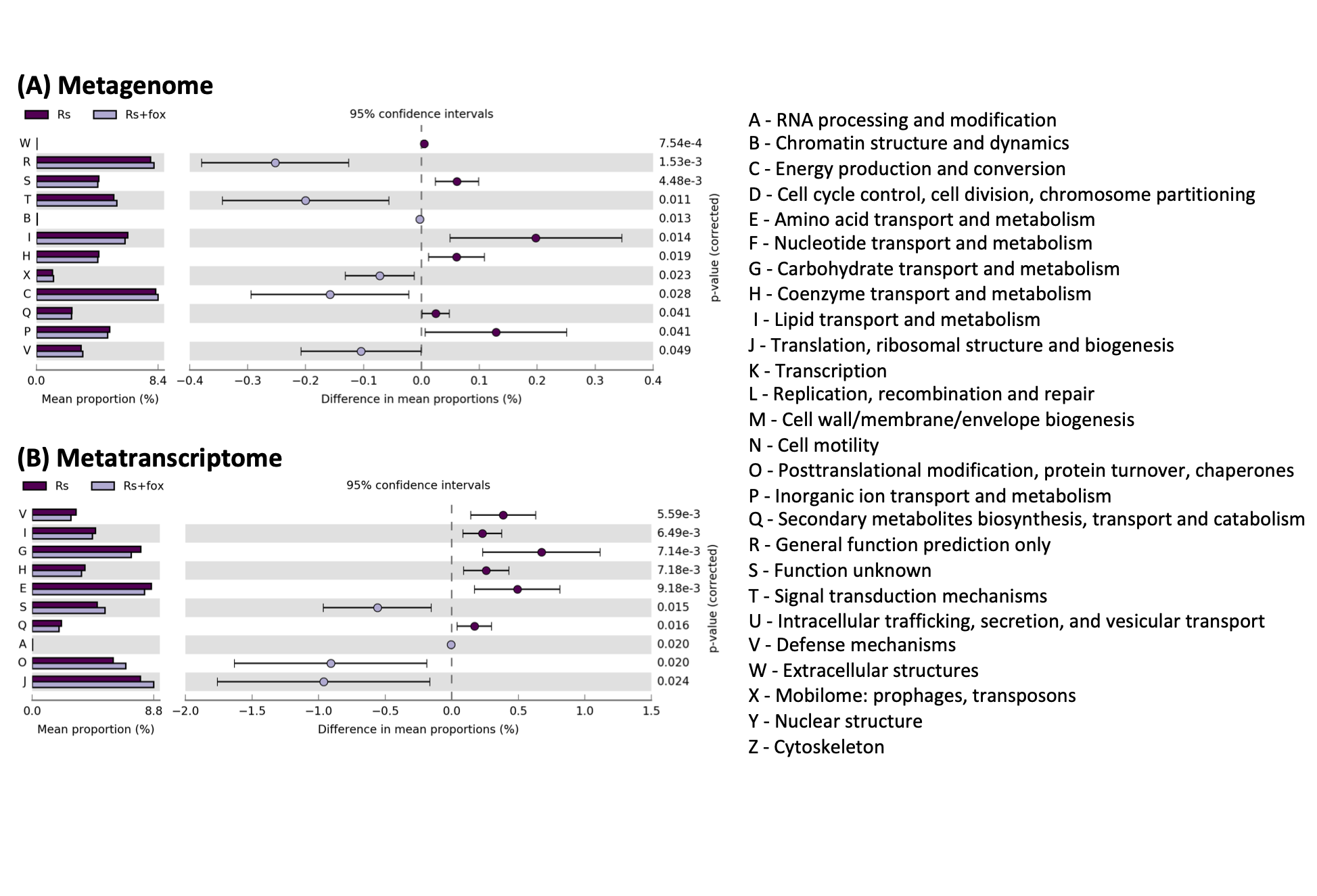
**

**Supplementary Figure 8.** Scatter-plot showing the differential abundance of sequences affiliated to bacterial functions in the *fox*-resistant cultivar after *fox* inoculation. The sequences were affiliated to functional categories based on COG database using **(A)** metagenome and **(B)** metatranscriptome datasets. P-values were calculated using Welch’s t-test with Benjamini-Hochberg correction (P < 0.05). A list with all the COG categories is shown in the right side of the figure.

**
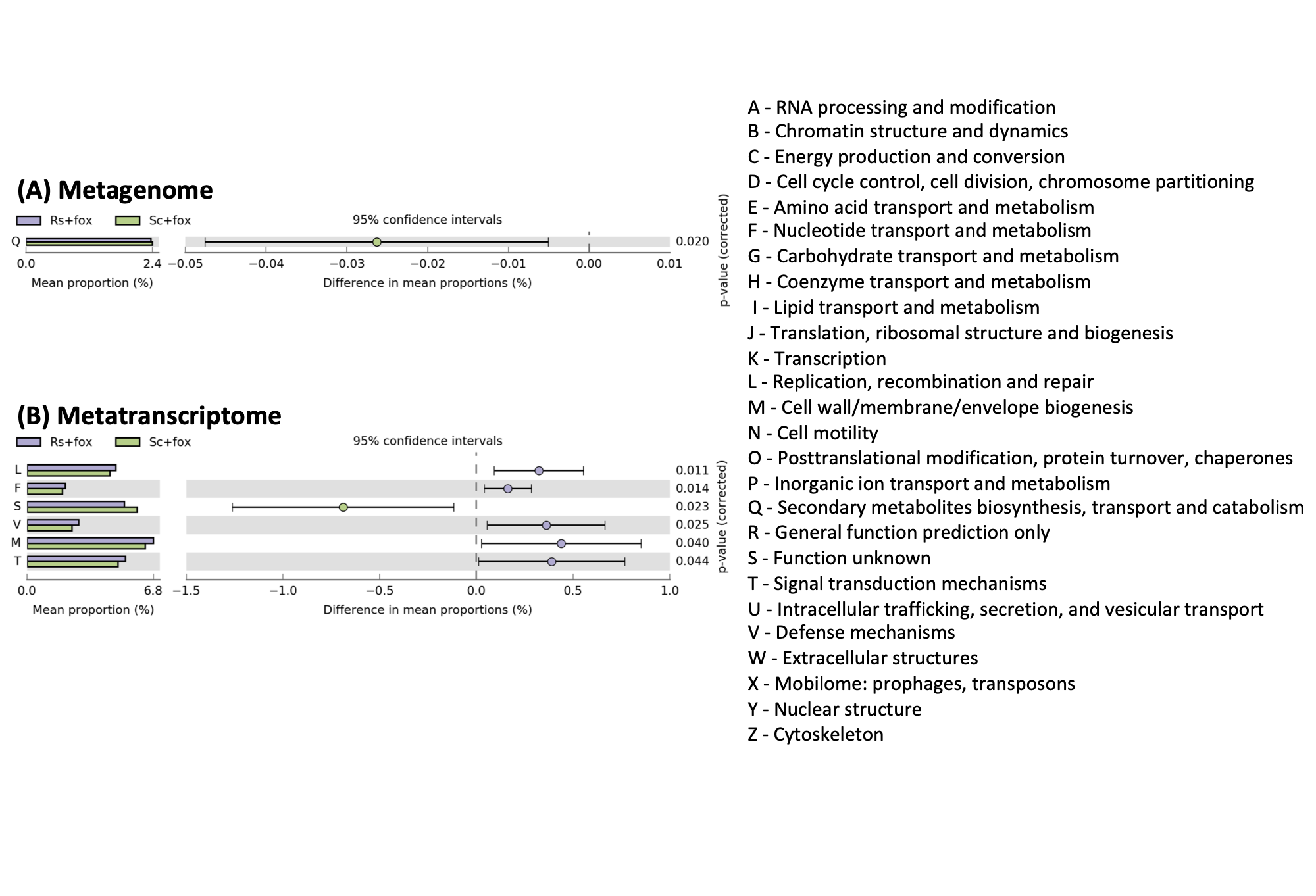
**

**Supplementary Figure 9.** Scatter-plot showing the differential abundance of sequences affiliated to bacterial functions comparing the *fox*-resistant cultivar with the susceptible after *fox* inoculation. The sequences were affiliated to functional categories based on COG database using **(A)** metagenome and **(B)** metatranscriptome datasets. P-values were calculated using Welch’s t-test with Benjamini-Hochberg correction (P < 0.05). A list with all the COG categories is shown in the right side of the figure.

**
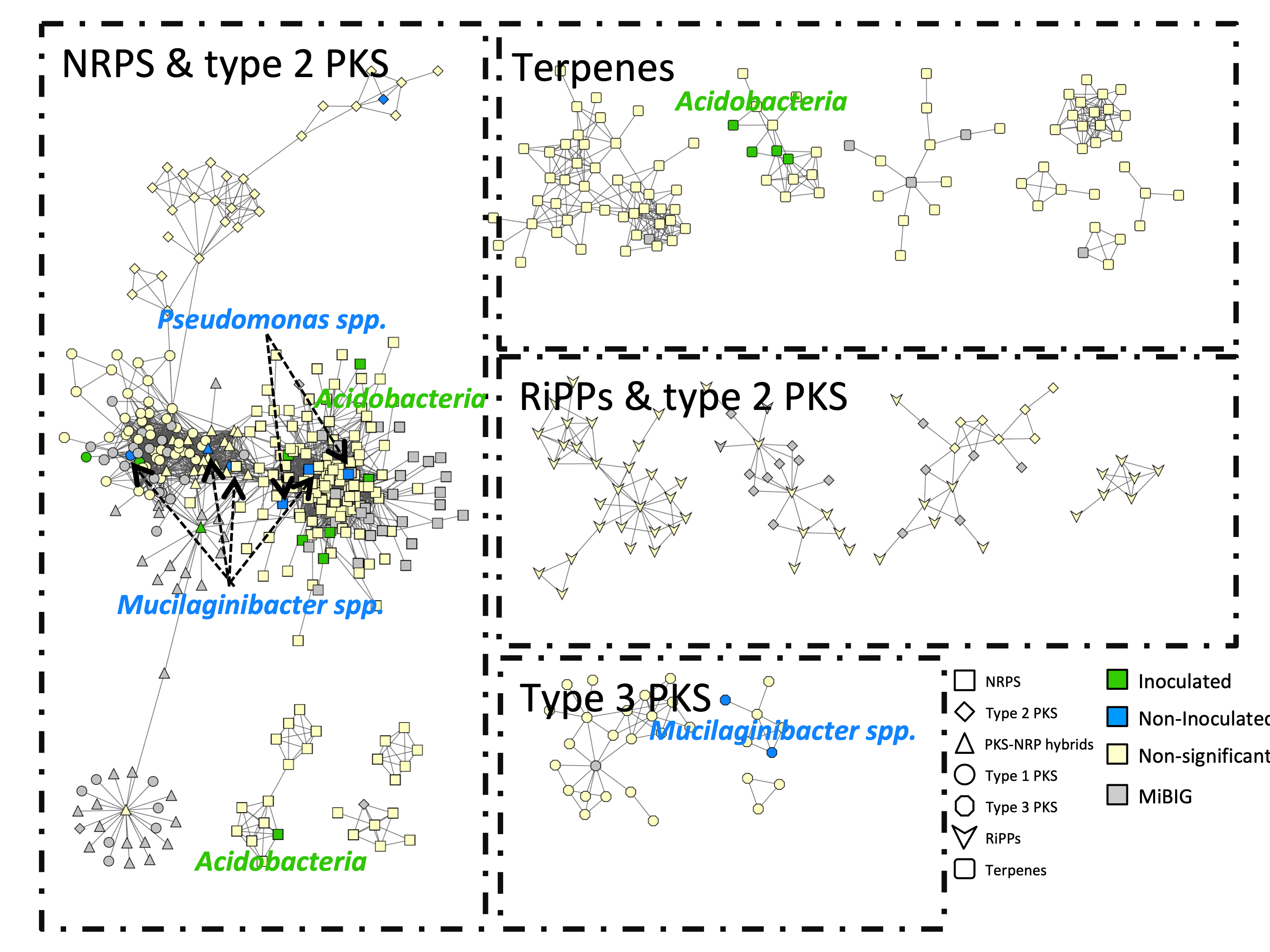
**

**Supplementary Figure 10.** Diversity and distribution of biosynthetic gene clusters in the rhizosphere microbiome of the susceptible common bean cultivar, inoculated and non-inoculated with *Fusarium* *oxysporum*. Sequence similarity network (constructed with BiG-SCAPE, threshold: 0.4) of the different classes of BGCs detected in the rhizosphere microbiome. Taxonomic assignment and BGC class annotation of the nodes are shown. Nodes with fewer than three connections were removed. Node colors represent statistical significance (FDR < 0.05): Yellow nodes are nonsignificant, green and blue nodes are significantly overrepresented in bean plants inoculated and non-inoculated with *fox.*


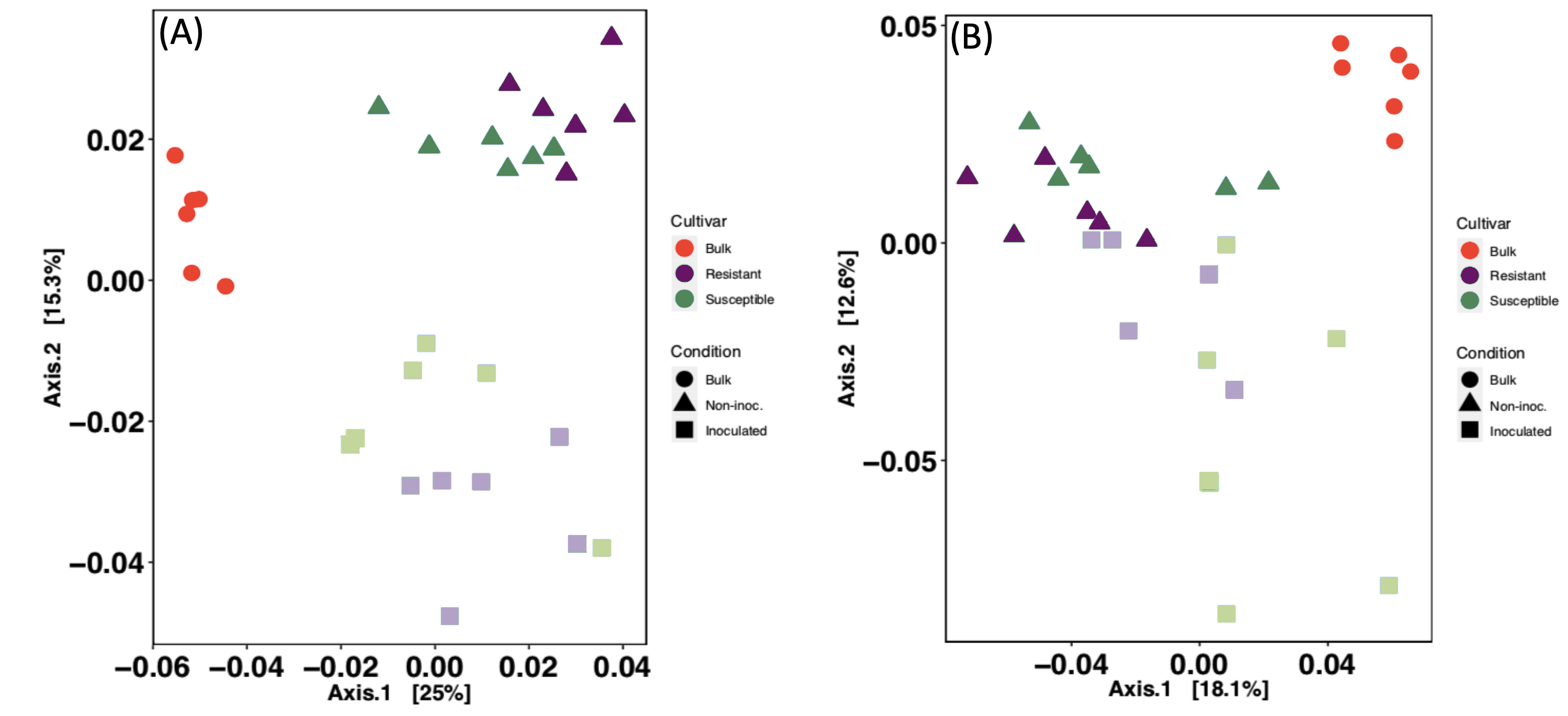


**Supplementary Figure 11.** Structure of the rhizopshere and bulk soil functional profile (based on Biosynthetic Gene Clusters) from two common bean cultivars non-inoculated or inoculated with *Fusarium* *oxysporum (fox)*. Principal coordinate analsyis (PCoA) comparing the functional profile structure in the rhizosphere of the two common bean cultivars after *fox* inoculation using **(A)** metagenome and **(B)** metatranscriptome.

**Supplementary Figure 12.** Multinomial species classification method (CLAM) for the niche occupancy test for the endosphere microbiome. The niche occupancy was evaluated in pairwise comparison between the treatments. The percentage of specialists is indicated in the graphs. R = *fox*-resistant cultivar; R*fox* = *fox*-resistant cultivar infected; S = susceptible cultivar; S*fox* – susceptible cultivar inoculated.

**
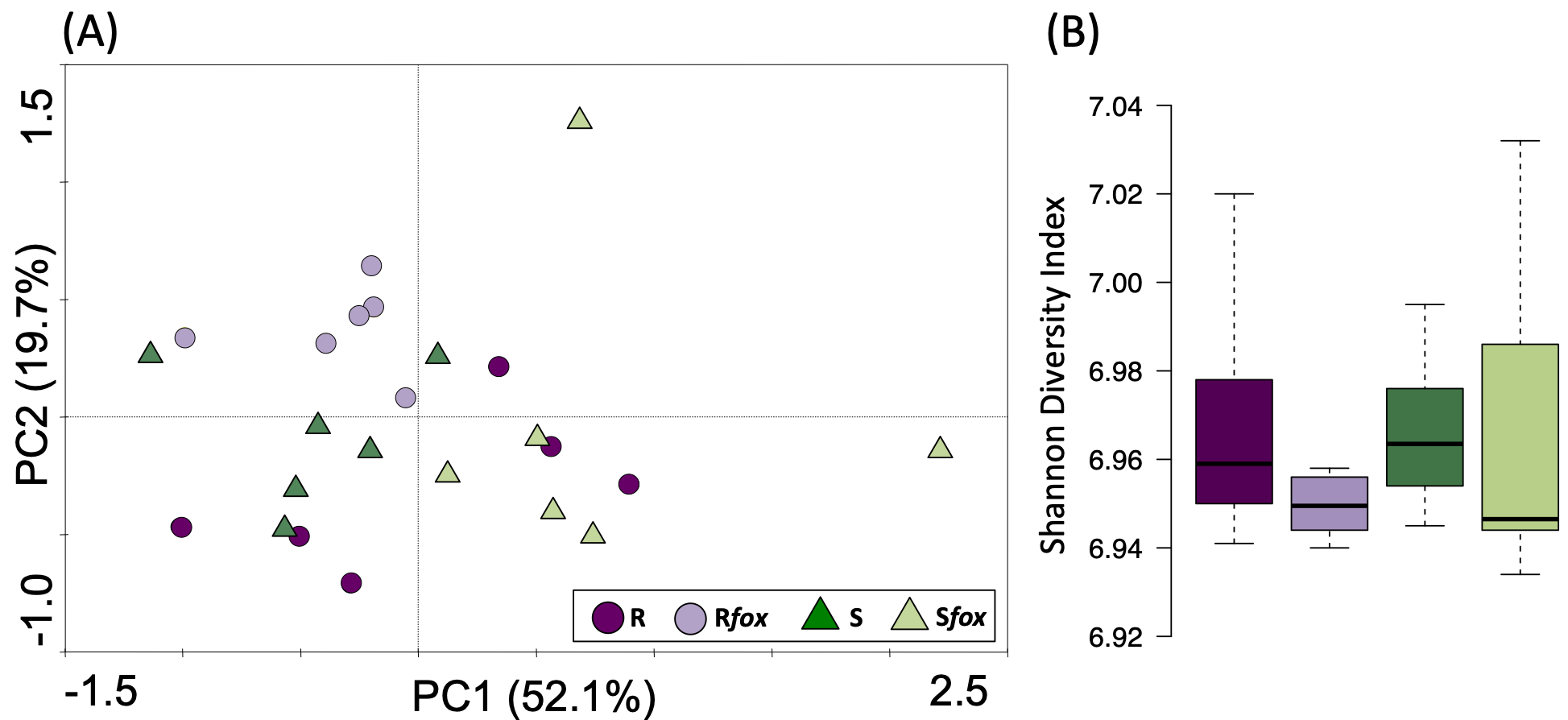
**

**Supplementary Figure 13.** Structure and diversity of endosphere functional profile (based on COG) from two common bean cultivars non-inoculated or inoculated with *Fusarium* *oxysporum (fox)*. **(A)** Principal component analsyis (PCA) comparing the functional profile structures using metagenome data. **(B)** Diversity measurements of the endosphere functional profile using metagenome. R = *fox*-resistant cultivar; R*fox* = *fox*-resistant cultivar infected; S = susceptible cultivar; S*fox* – susceptible cultivar infected.


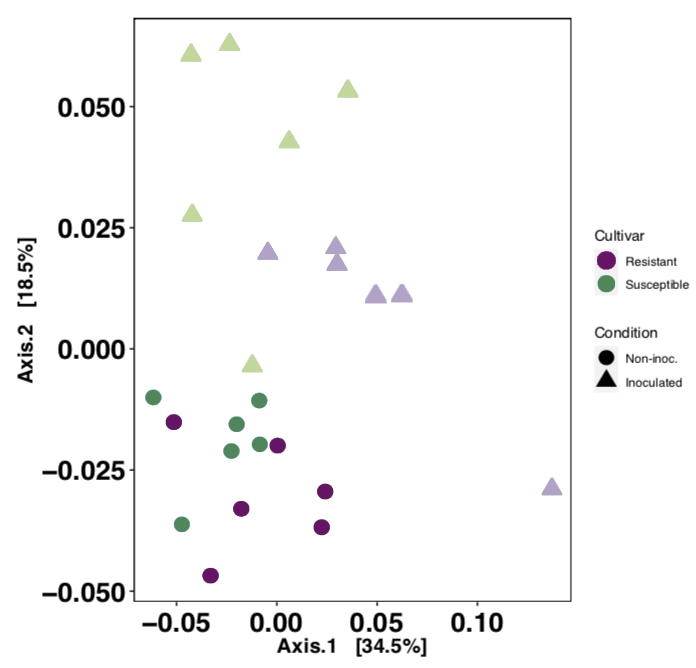


**Supplementary Figure 14.** Principal coordinate analsyis (PCoA) comparing the endosphere functional profile structure (based on Biosynthetic Gene Clusters) in the rhizosphere of the two common bean cultivars non-inoculated or inoculated with *Fusarium* *oxysporum (fox)* using metagenome data.

**
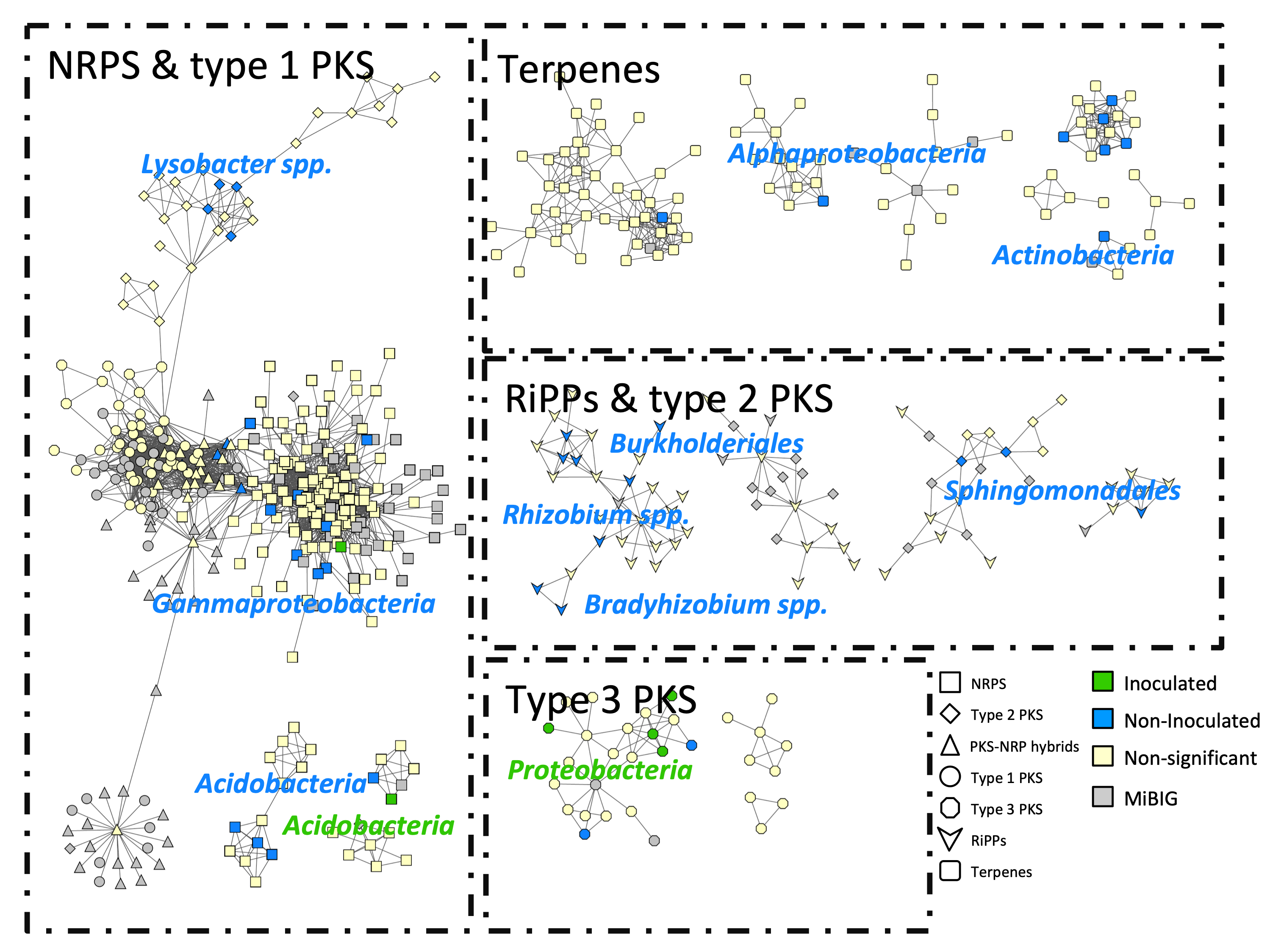
**

**Supplementary Figure 15.** Diversity and distribution of biosynthetic gene clusters in the endosphere microbiome of the resistant common bean cultivar, non-inoculated or inoculated with *Fusarium* *oxysporum* (*fox*). Sequence similarity network (constructed with BiG-SCAPE, threshold: 0.4) of the different classes of BGCs detected in the rhizosphere microbiome. Taxonomic assignment and BGC class annotation of the nodes are shown. Nodes with fewer than three connections were removed. Node colors represent statistical significance (FDR < 0.05): Yellow nodes are nonsignificant, green and blue nodes are significantly overrepresented in bean plants inoculated and non-inoculated with *fox*.

**
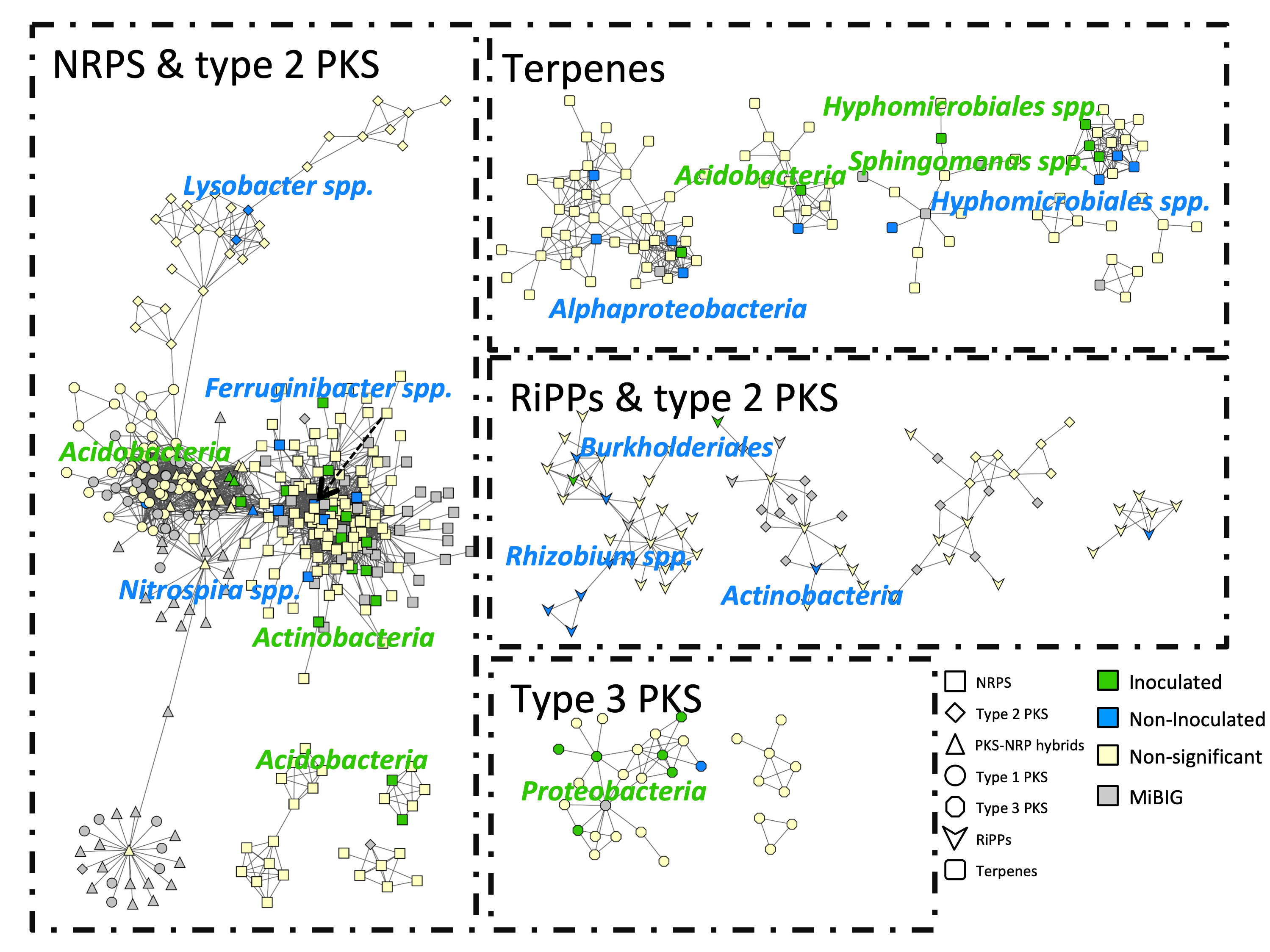
**

**Supplementary Figure 16.** Diversity and distribution of biosynthetic gene clusters in the endosphere microbiome of the susceptible common bean cultivar, non-inoculated or inoculated with *Fusarium* *oxysporum* (*fox*). Sequence similarity network (constructed with BiG-SCAPE, threshold: 0.4) of the different classes of BGCs detected in the rhizosphere microbiome. Taxonomic assignment and BGC class annotation of the nodes are shown. Nodes with fewer than three connections were removed. Node colors represent statistical significance (FDR < 0.05): Yellow nodes are nonsignificant, green and blue nodes are significantly overrepresented in bean plants inoculated and non-inoculated with *fox*.
